# Global research trends of diabetes remission: a bibliometric study

**DOI:** 10.3389/fendo.2023.1272651

**Published:** 2023-11-28

**Authors:** Xue Yang, Zhiwei He, Qilin Chen, Yu Chen, Guofang Chen, Chao Liu

**Affiliations:** ^1^ Department of Endocrinology, Affiliated Hospital of Integrated Traditional Chinese and Western Medicine, Nanjing University of Chinese Medicine, Nanjing, China; ^2^ Jiangsu Province Academy of Traditional Chinese Medicine, Nanjing, China; ^3^ KweiChow Moutai Hospital, Renhuai, China; ^4^ School of Traditional Chinese and Western Medicine, Gansu University of Chinese Medicine, Lanzhou, China

**Keywords:** diabetes mellitus, remission, bibliometric study, Citespace, VOSviewer

## Abstract

**Background:**

Research on diabetes remission has garnered prominence in recent years. However, to date, no pertinent bibliometric study has been published. This study sought to elucidate the current landscape and pinpoint potential new research directions through a bibliometric analysis of diabetes remission.

**Methods:**

We perused relevant articles on diabetes remission from January 1, 2000, to April 16, 2023, in the Web of Science. We utilized CiteSpace software and VOSviewer software to construct knowledge maps and undertake analysis of countries, institutional affiliations, author contributions, journals, and keywords. This analysis facilitated the identification of current research foci and forecasting future trends.

**Results:**

A total of 970 English articles were procured, and the annual publication volume manifested a steady growth trend. Most of the articles originated from America (n=342, 35.26%), succeeded by China and England. Pertaining to institutions, the University of Newcastle in England proliferated the most articles (n=36, 3.71%). Taylor R authored the most articles (n=35, 3.61%), and his articles were also the most co-cited (n=1756 times). *Obesity Surgery* dominated in terms of published articles (n=81, 8.35%). “Bariatric surgery” was the most prevalently used keyword. The keyword-clustering map revealed that the research predominantly centered on diabetes remission, type 1 diabetes, bariatric surgery, and lifestyle interventions. The keyword emergence and keyword time-zone maps depicted hotspots and shifts in the domain of diabetes remission. Initially, the hotspots were primarily fundamental experiments probing the feasibilities and mechanisms of diabetes remission, such as transplantation. Over the course, the research trajectory transitioned from basic to clinical concerning diabetes remission through bariatric surgery, lifestyle interventions, and alternative strategies.

**Conclusion:**

Over the preceding 20 years, the domain of diabetes remission has flourished globally. Bariatric surgery and lifestyle interventions bestow unique advantages for diabetes remission. Via the maps, the developmental milieu, research foci, and avant-garde trends in this domain are cogently portrayed, offering guidance for scholars.

## 1 Introduction

In recent years, the incidence of diabetes has escalated precipitously (1, 2), and its detriment to humans surpasses that of cardiovascular diseases, ascertaining its position first among the world’s top ten chronic diseases (3). Diabetes inflicts considerable physiological (4–7), psychological (8–10), and economic (3) adversities to patients. The cognizance rate of diabetes remains low (11), and an increasing number of young individuals are succumbing to it (12). Thus, it is imperative to elucidate effective methods for the prevention and treatment of diabetes.

Historically, diabetes has been perceived as an intractable and perpetual ailment necessitating the prolonged administration of hypoglycemic agents. However, concomitant with the evolution in medical technology and the amassment of pertinent evidence-based findings, the remission of diabetes is progressively being substantiated (13). A plethora of studies have illuminated the salutary effects of diabetes remission, especially the remission of type 2 diabetes mellitus (T2DM), and have realized monumental accomplishments in strategies, mechanisms, and predictive indices (14–16). “Remission” denotes that, via certain interventions, diabetes patients can sustain glycated hemoglobin A1c (HbA1c) levels, or blood glucose levels, at normative or near-normative thresholds absent the deployment of hypoglycemic agents, for a duration exceeding a specified span (17). One investigation revealed that from pre-diabetes to manifest diabetes, the risk of cardiovascular pathology in patients augmented to an approximate 23% (18). Furthermore, subsequent to the remission of diabetes, not solely did the metrics pertinent to glucose and lipid metabolism exhibit marked amelioration, but the cardiovascular disease risk plummeted to 7%, whilst the probability of other chronic sequelae such as diabetic foot notably diminished (18). Hence, the remission of diabetes bears profound relevance. Over preceding decades, a myriad of studies on diabetes remission has been disseminated. Yet, no study has holistically assessed the associated publications.

Bibliometrics empower the application of mathematical and statistical techniques to quantitatively scrutinize a multitude of articles within a designated research domain, thereby unveiling its research status and trajectories. CiteSpace and VOSviewer constitute the preeminent literature information visualization software tools, extensively employed across diverse disciplines such as medicine, science and technology, economic governance, jurisprudence, agronomy, and humanities (19–24). Within the realm of metabolic disorders, a multitude of researchers have harnessed this methodology to assess their investigative content (25–30). However, as of this juncture, there exists no distinct scholarly metric examination of knowledge schematics pertaining to diabetes remission. This study appraised articles on diabetes remission promulgated from January 1, 2000, to April 16, 2023. Our objective is to delineate the extant paradigm in this domain and ascertain emergent research vectors.

## 2 Materials and methods

### 2.1 Data collection

On April 16, 2023, the two authors (ZH and QC) independently undertook searches and accrued articles from the Web of Science database, probing for articles bearing the title “diabetes remission” or “diabetes mellitus remission” or “type 2 diabetes remission” or “type 2 diabetes mellitus remission” or “T2DM remission” or “remission of diabetes” or “remission of diabetes mellitus” or “remission of type 2 diabetes” or “remission of type 2 diabetes mellitus” or “reversal of diabetes” or “reversal of diabetes mellitus” or “reversal of type 2 diabetes” or “reversal of type 2 diabetes mellitus” or “reversal of T2DM” or “reverse diabetes” or “reverse diabetes mellitus” or “reverse type 2 diabetes” or “ reverse type 2 diabetes mellitus” or “ reverse T2DM” or “diabetes be reversed” or “diabetes mellitus be reversed” or “type 2 diabetes be reversed” or “type 2 diabetes mellitus be reversed” or “T2DM be reversed” spanning from January 1, 2000, to April 16, 2023, in the English language. Post exclusion of news reports, conference abstracts, health science dissemination, and articles that were incongruous to the theme, salient data (titles, keywords, author metadata, abstracts, citations, etc.) was retrieved and archived in TXT format for subsequent utilization. Thereafter, two authors (GC and CL) omitted articles that were inapposite to the central theme, culminating in a total of 970 references.

### 2.2 Statistical methods

Initially, we fabricated cooperation network maps of nations, institutions, and contributors utilizing CiteSpace (6.1.R6 Advanced). Within these maps, each node signifies a distinct nation, institution, or contributor, and the connectors imply that the associated nodes have collaborative affiliations. The more pronounced the connectors, the more robust the collaborative affiliations. Subsequently, we incepted keyword co-occurrence and keyword emergence maps. We then executed a keyword clustering exploration: a clustering map, a chronology map, and a temporal sector map. The magnitude of the circle within the map corresponds to the recurrence: the more expansive the circle’s diameter, the more elevated the recurrence of the node. The breadth of the circle depicts the recurrence of the associated content manifested across distinct years, and the links between nodes denote co-presence. The collaborative network maps generated by CiteSpace lack the capability to delineate the dimensions of the nodes; hence, they are unable to delineate variances in magnitude. Additionally, a multitude of labels, such as nations, institutions, and authors, often induce overlap, diminishing the visual clarity. The collaborative network map fashioned by VOSviewer possesses the merit of succinctness and lucidity (31). As such, we harnessed VOSviewer (1.6.18) to conduct a concurrent analysis of nations, institutions, authorship, and collaborations in articles, and to instigate a co-presence examination of co-citations and article sources. The circles on the map represent distinct nations, institutions, authors, and journals, and the size of the circle delineates quantity. The more expansive the circle’s diameter, the greater the node count. The connector extending between the circles indicates a co-presence association amongst nodes.

## 3 Results and analysis

### 3.1 Analysis of the source of the article’s publication

#### 3.1.1 Annual growth trend of publications

**Figure 1:**
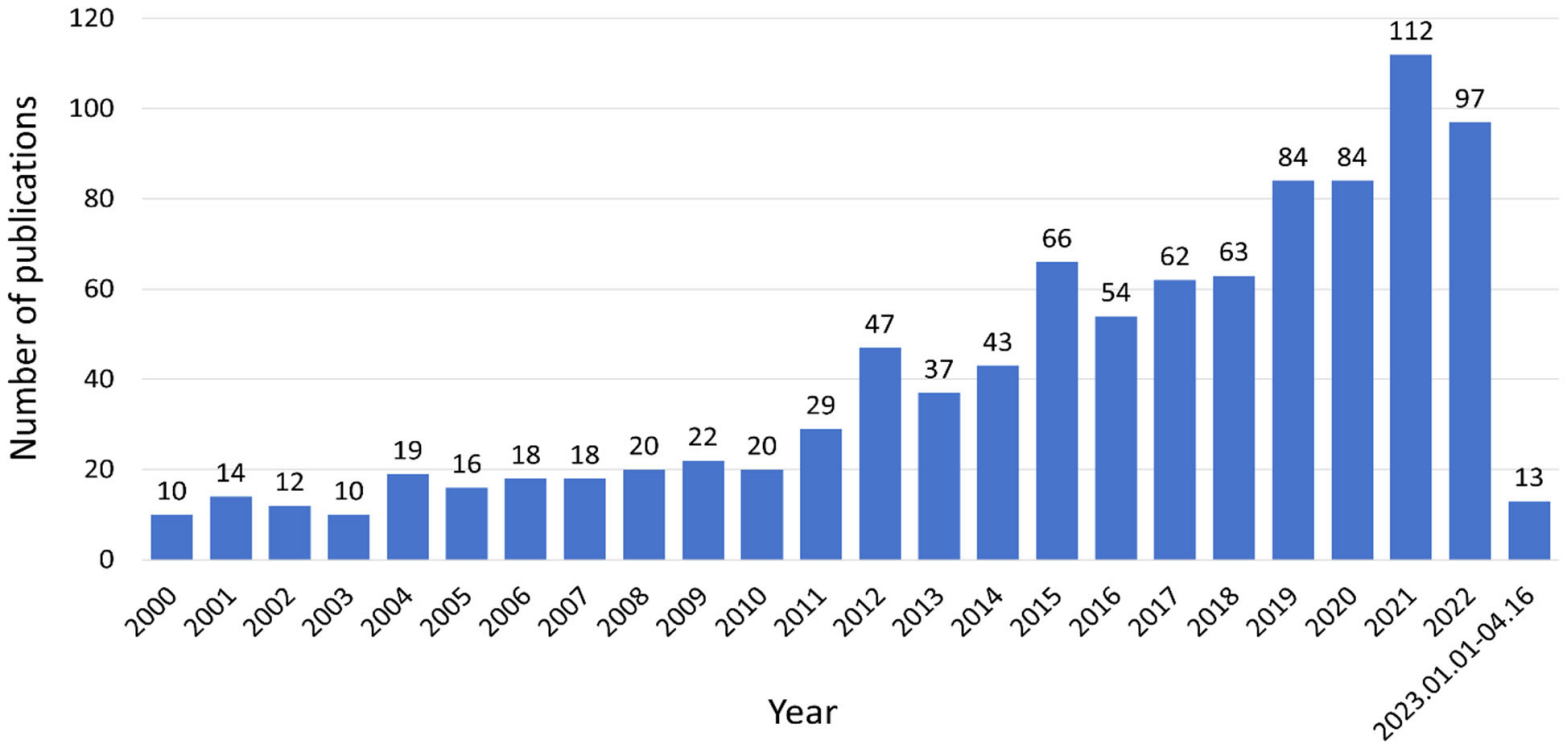
Annual publications worldwide.

Based on the distribution of the 970 included articles by publication time (**Figure 1**), the articles pertaining to diabetes remission exhibited a progressive upward trend from January 1, 2000, to April 16, 2023. Notably, post-2010, this escalation became even more pronounced. The volume of articles published peaked at 112 in 2021 and experienced a marginal decline to 97 by 2022. Overall, research in the realm of diabetes remission has garnered increasing scholarly attention.

#### 3.1.2 Countries analysis

**Figure 2:**
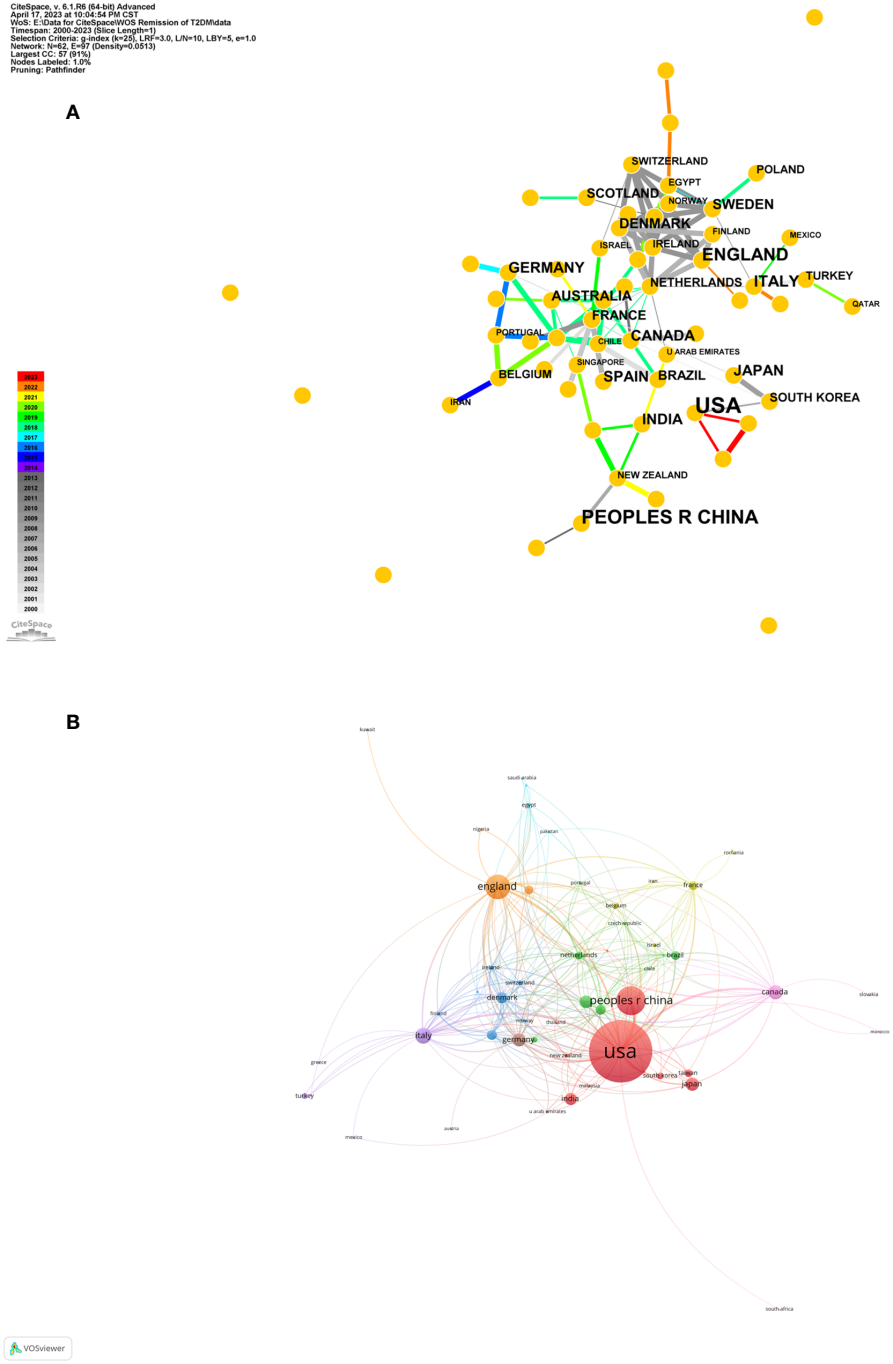
Country cooperation network map. **(A)** Country cooperation network map made by CiteSpace. The node represents the certain country while the links reflect the co-occurrence relationships. The color of node and line indicates different years. **(B)** Country cooperation network map made by VOSviewer. The size of node indicates the number of articles published by a certain country, and the links between two nodes mean a collaboration between each other.

A cumulative of 790 articles were gleaned from 63 countries, and we selected the top ten countries based on their publication output for the publication scale (**Table 1**). America had the preeminent publication volume (n=342, 35.26%), trailed by China (n=130, 13.40%) and England (n=108, 11.13%). A topological representation of inter-country collaboration is delineated in **Figure 2**. **Figure 2B** underscores the America’s dominant publication activity, while **Figure 2A** highlights the symbiotic scholarly activities primarily among European nations.

**Table 1:** Top 10 countries in the number of published articles.

Rank	Country	Count	Percent
1	America	342	35.26%
2	China	130	13.40%
3	England	108	11.13%
4	Italy	61	6.29%
5	Canada	52	5.36%
6	Germany	48	4.95%
7	Japan	47	4.85%
8	Spain	45	4.64%
9	India	43	4.43%
10	Denmark	38	3.92%

#### 3.1.3 Institutions analysis

**Figure 3:**
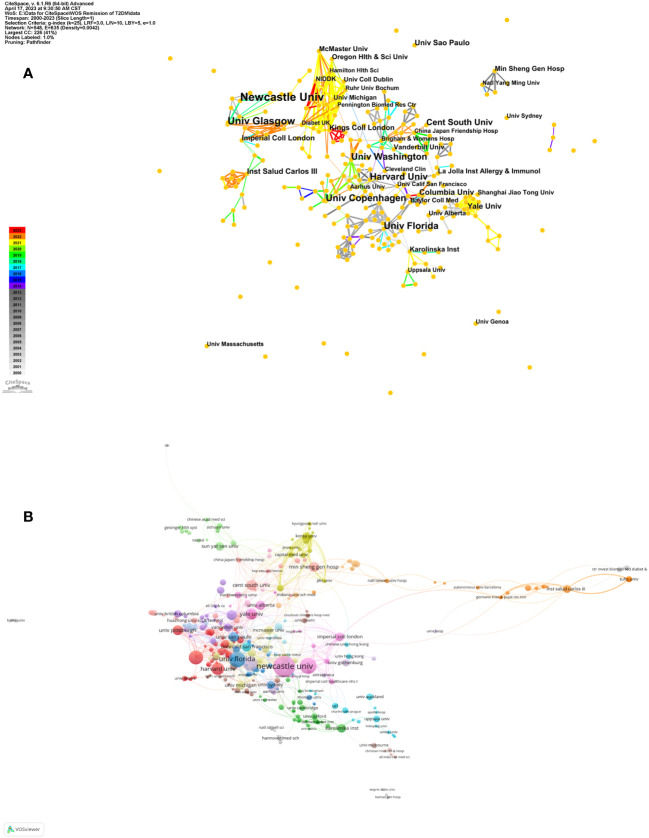
Institution cooperation network map. **(A)** Institution cooperation network map made by CiteSpace. The node represents the certain institution while the links reflect the co-occurrence relationships. The color of node and line indicates different years. **(B)** Institution cooperation network map made by VOSviewer. The size of node indicates the number of articles published by a certain institution, and the links between two nodes mean a collaboration between each other.

These articles were disseminated by 1,568 institutions. The foremost institutions in terms of publication volume are cataloged in **Table 2**. Evidently, Newcastle University in England leads with the maximum number of articles (n=36, 3.71%), succeeded by Harvard University in America (n=33, 3.40%), and the State University System of Florida in America (n=30, 3.09%). Half of the top ten institutions are American, collectively contributing 134 articles, which constitutes 50.76% of the articles from these leading institutions. Inter-institutional collaboration networks are depicted in **Figure 3**. **Figure 3A** elucidates the extensive synergy among institutions, with Newcastle University’s predominant partnership being with the University of Glasgow. **Figure 3B** accentuates the robust collaborative network among American institutions.

**Table 2:** Top 10 institutions in the number of published articles.

Rank	Institutions	Count	Percent
1	Newcastle University - England	36	3.71%
2	Harvard University - America	33	3.40%
3	State University System of Florida - America	30	3.09%
4	University of California System - America	25	2.58%
5	University of Copenhagen - Denmark	25	2.58%
6	University of Glasgow - England	25	2.58%
7	University of Florida - America	24	2.47%
8	University of London - England	23	2.37%
9	National Institutes of Health - America	22	2.27%
10	Network Biomedical Research Center - Spain	21	2.16%

#### 3.1.4 Authors and co-cited authors analysis

**Figure 4:**
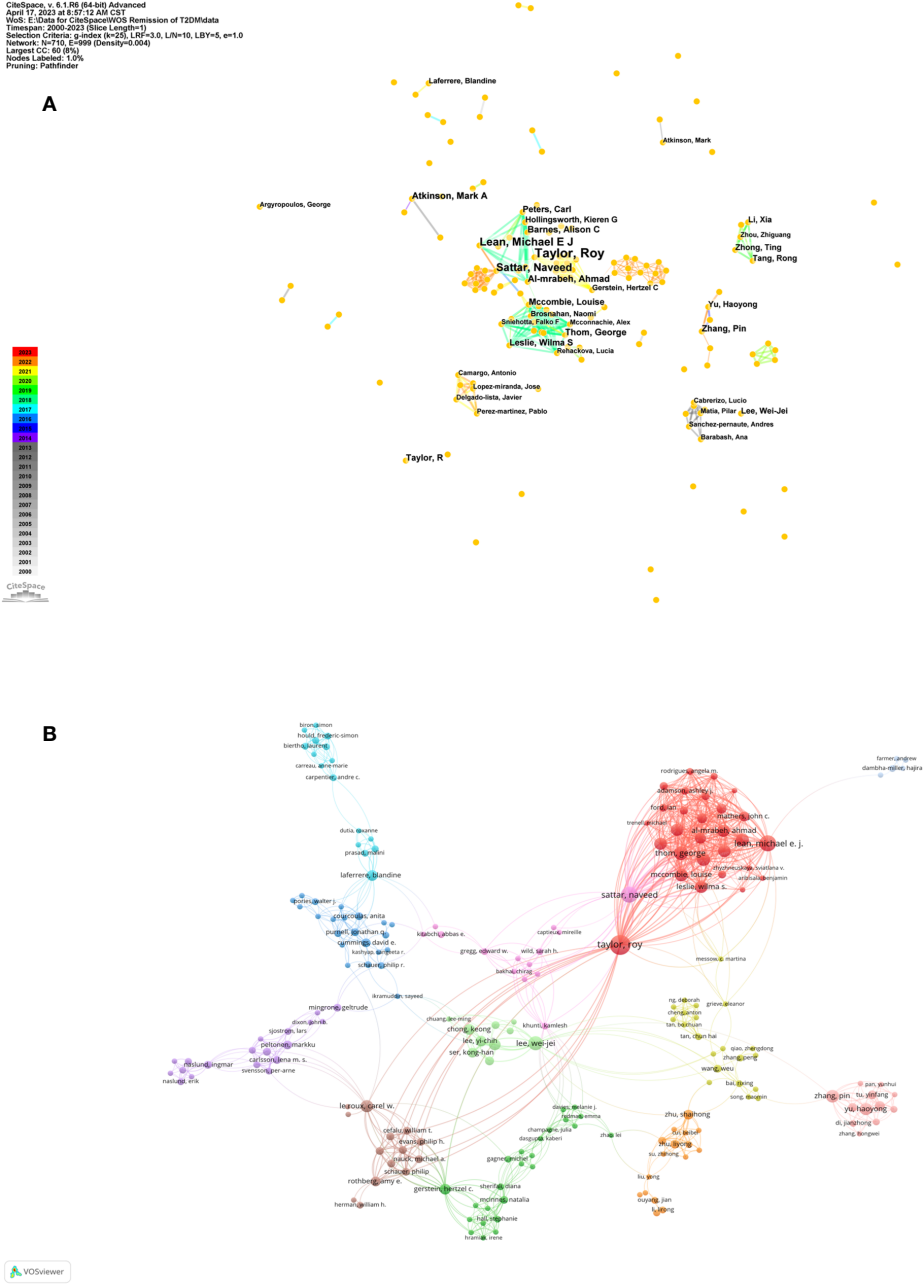
Author cooperation network map. **(A)** Author cooperation network map made by CiteSpace. The node represents the certain author while the links reflect the co-occurrence relationships. The color of node and line indicates different years. **(B)** Author cooperation network map made by VOSviewer. The size of node indicates the number of articles published by a certain author, and the links between two nodes mean a collaboration between each other.

A staggering 5,697 authors contributed to these articles. The top ten prolific authors are enumerated in **Table 3**. The triumvirate of authors with the most substantial publication contributions are Taylor R (n=35, 3.61%), Lean MEJ (n=19, 1.96%), and Sattar N (n=17, 1.75%). As depicted in **Figure 4A**, the nexus between authors from Taylor R’s contingent at Newcastle University in the UK is particularly strong. Moreover, Taylor R’s assemblage manifests significant collaboration with Lean MEJ’s and Sattar N’s teams at the University of Glasgow. **Figure 4B** reveals the presence of several research consortia dedicated to diabetes remission. Taylor R, apart from being the most published author, also exhibits extensive inter-team collaborations. Additionally, authors like Lee WJ, Gerstein HC, and le Roux CW exhibit notable interdisciplinary collaborations.

**Table 3:** Top 10 authors in the number of published articles.

Rank	Author	Count	Percent	Institutions
1	Taylor R	35	3.61%	Newcastle University - England
2	Lean MEJ	19	1.96%	University of Glasgow - England
3	Sattar N	17	1.75%	University of Glasgow - England
4	Zhang P	13	1.34%	Shanghai Jiao Tong University - China
5	Barnes AC	12	1.24%	Newcastle University - England
6	Le Roux CW	12	1.24%	University College Dublin - Ireland
7	Leslie WS	12	1.24%	University of Glasgow - England
8	Mccombie L	12	1.24%	University of Glasgow - England
9	Thom G	12	1.24%	University of Glasgow - England
10	Lee WJ	11	1.13%	Min-Sheng General Hospital - China

**Figure 5:**
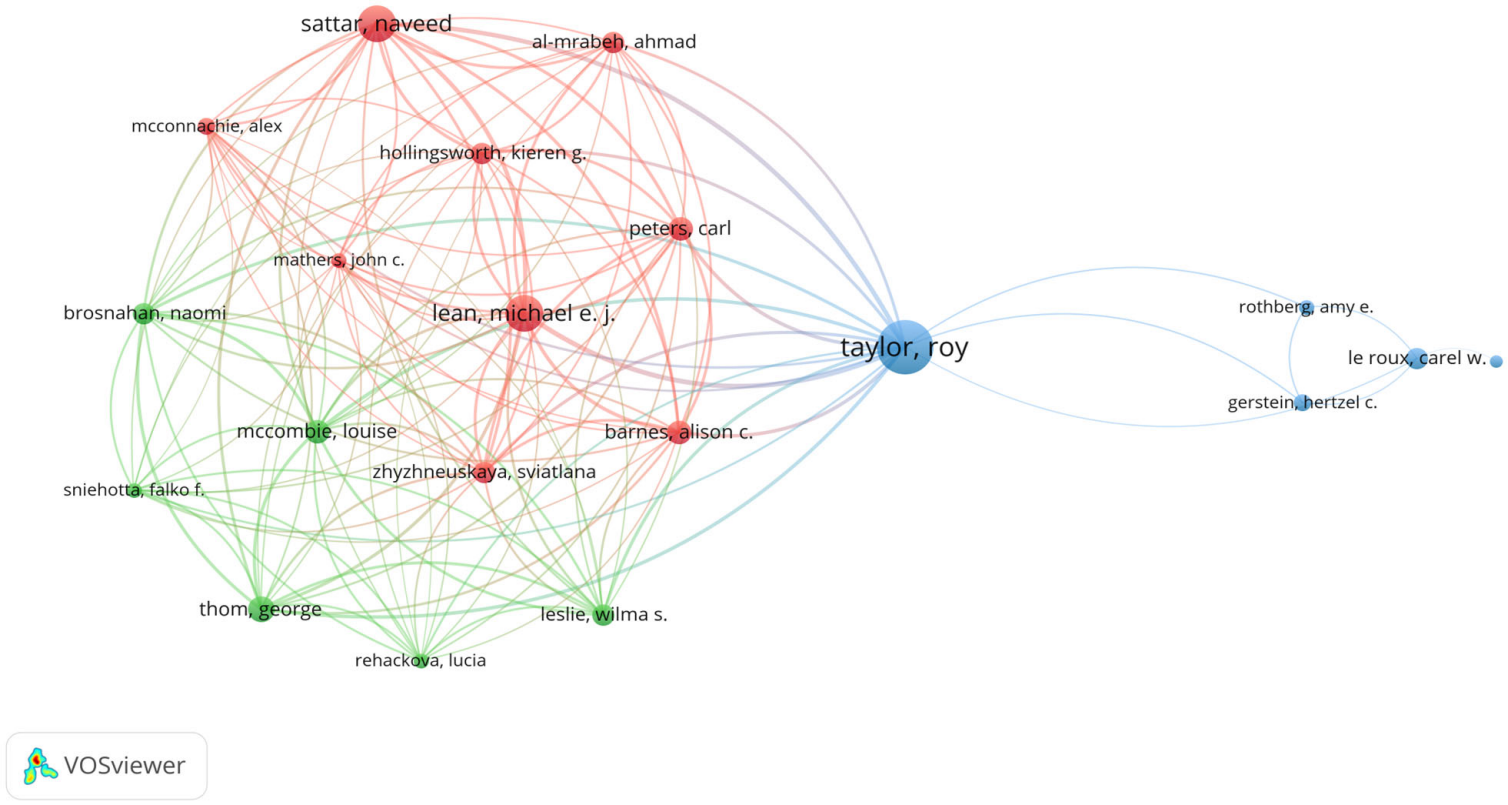
Author co-citation network map. The size of node indicates the co-cited frequency of a certain author, and the links between two circles mean a co-citation relationship between authors.

When two or more authors are concurrently cited in a separate article, a co-citation relationship materializes. As presented in **Table 4**, 66 authors have been co-cited more than five times. The three authors with the highest co-citation frequencies are Taylor R (1,756 times), Lean MEJ (1,569 times), and Sattar N (1,593 times), attesting to the wide recognition and impact of their scholarship in the diabetes remission domain. **Figure 5** showcases the co-citation networks of the top 20 authors, underscoring the pervasive co-citation relationships among them.

**Table 4:** Top 10 authors in the number of co-citation.

Rank	Author	Articles	Co-citations	Total linkstrength
1	Taylor R	24	1756	147
2	Lean MEJ	16	1569	120
3	Sattar N	16	1593	117
4	Peters C	10	1523	102
5	Thom G	11	1283	102
6	Barnes AC	10	1449	99
7	Mccombie L	10	1291	99
8	Zhyzhneusk S	9	1438	98
9	Leslie WS	9	1272	96
10	Hollingsworth KG	9	1295	89

#### 3.1.5 Journals analysis

**Figure 6:**
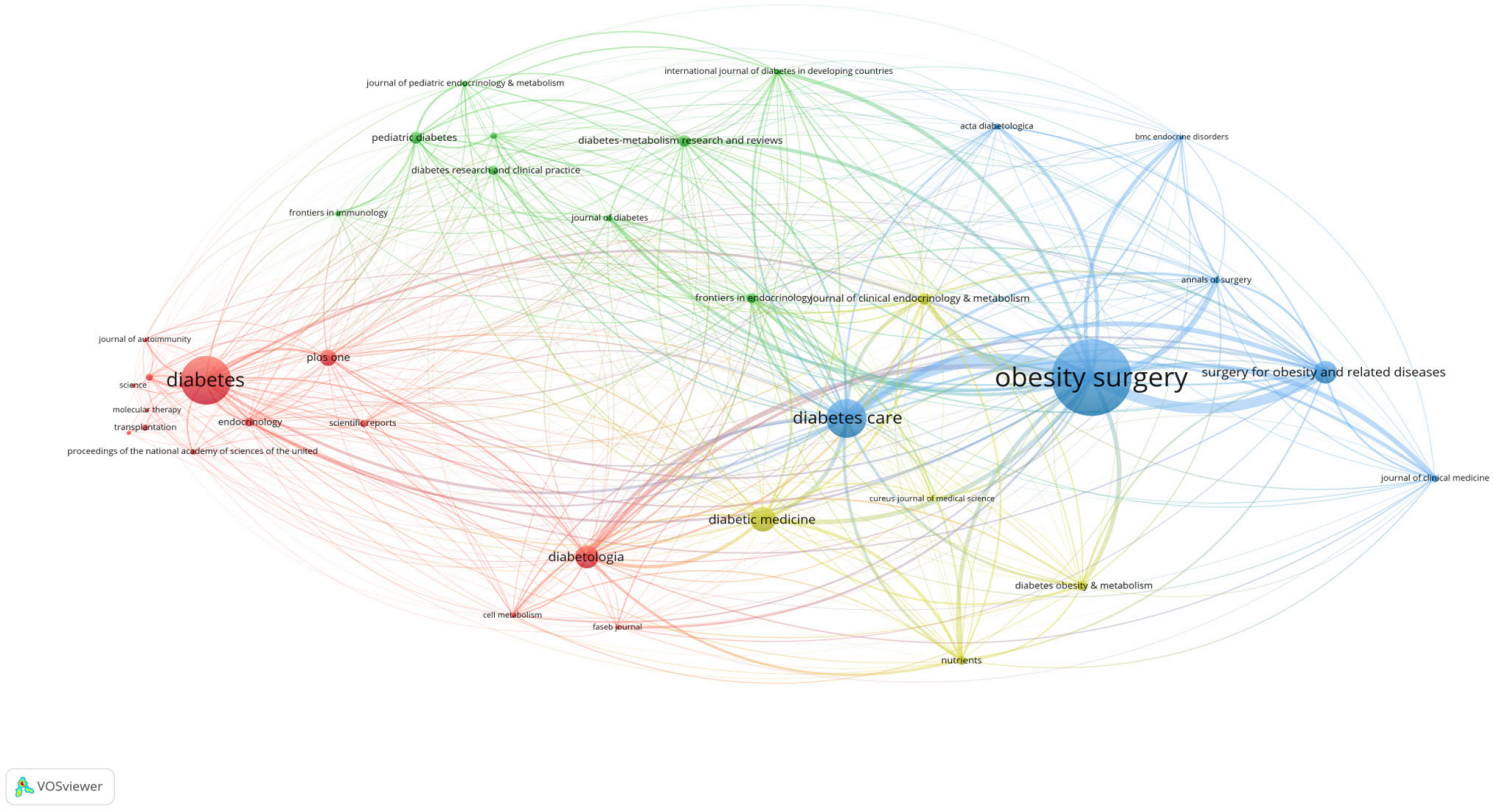
Journal cooperation network map. The size of node indicates the number of articles published by a certain journal, and the links between two nodes mean a collaboration between each other.

**Figure 7:**
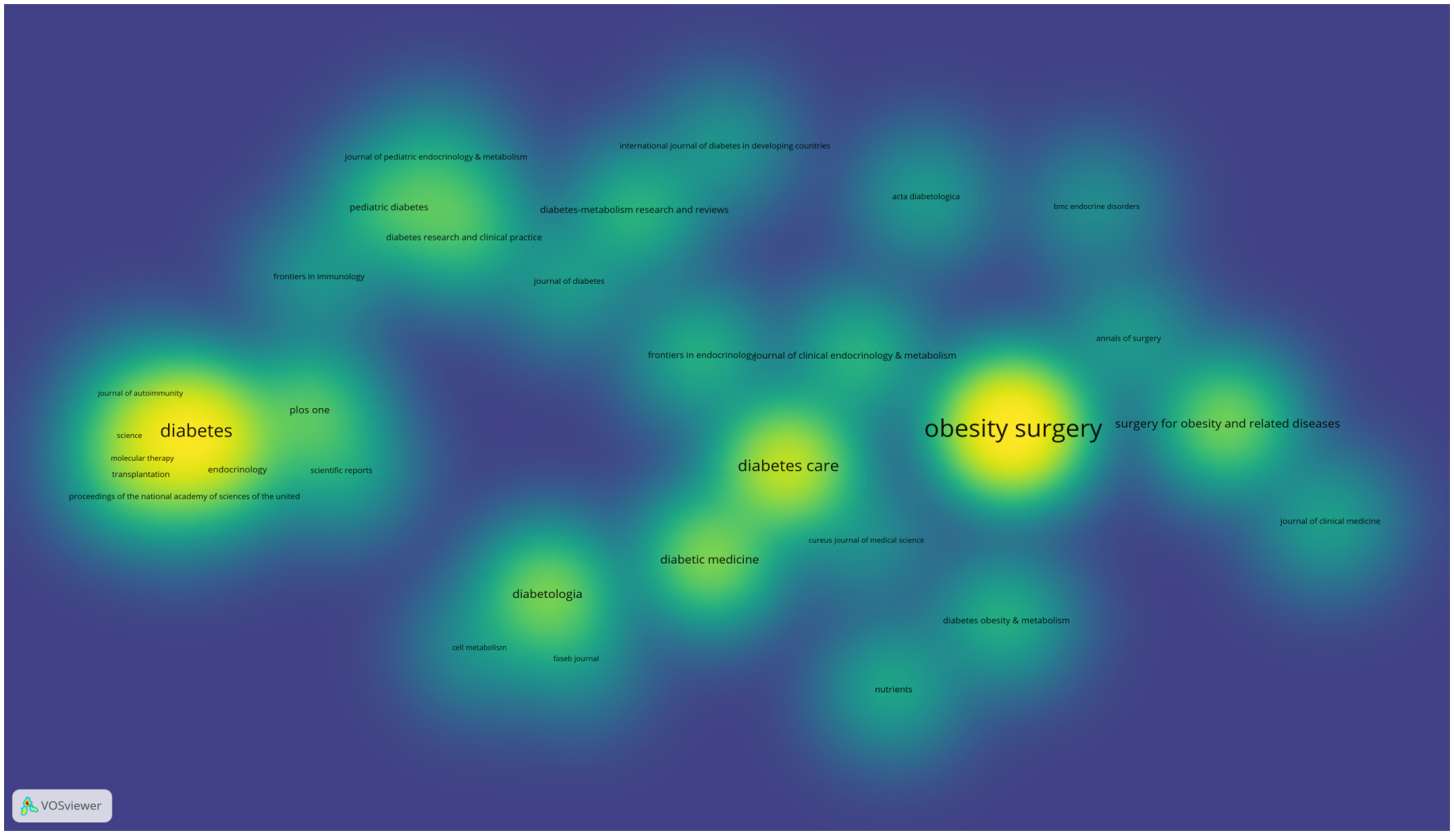
Journal cooperation density map. The size of the word and round, and the opacity of yellow are positively associated with the cited frequency.

In aggregate, 790 articles were procured from 379 journals. **Table 5** enumerates the top ten journals by publication count, with *Obesity Survey* (n=81, 8.35%), *Diabetes* (n=51, 5.26%), and *Diabetes Care* (n=42, 4.33%) predominating. Among the top 10 journals (**Table 5**), 50% (5/10) originated from America, succeeded by 30% (3/10) from England. The journal impact metric for *Diabetes Care* was the most notable (JIF2022 = 16.2, JCI2022 = 3.69), with *Diabetologia* following (JIF2022 = 8.2, JCI2022 = 1.83). **Figure 6** depicts the collaborative network amongst journals disseminating research articles pertinent to diabetes remission. **Figure 7** offers a density map, highlighting the predilection of different journals within the context of diabetes remission.

**Table 5:** Top 10 journals that publish research articles.

Rank	Journal	Count	Percent	Country	JIF (2022)	JCI (2022)	JCR (2022)
1	*Obesity Surgery*	81	8.35%	America	2.9	1.23	Q2
2	*Diabetes*	51	5.26%	America	7.7	1.68	Q1
3	*Diabetes Care*	42	4.33%	America	16.2	3.69	Q1
4	*Diabetic Medicine*	26	2.68%	England	3.5	0.71	Q3
5	*Diabetologia*	24	2.47%	Germany	8.2	1.83	Q1
6	*Surgery for Obesity and Related Diseases*	24	2.47%	Netherlands	3.1	1.35	Q1
7	*Plos One*	16	1.65%	America	3.7	0.91	Q2
8	*Pediatric Diabetes*	13	1.34%	England	3.4	1.01	Q3
9	*Diabetes Metabolism Research and Reviews*	12	1.24%	England	8	1.42	Q1
10	*Journal of Clinical Endocrinology and Metabolism*	12	1.24%	America	5.8	1.27	Q1

#### 3.1.6 Analysis of cited articles

Gleaning from the citation frequency table (**Table 6**), the paramountly cited article was penned by Kieffer et al., titled “Reverse of diamonds with insulin-producing cells derived *in vitro* from human pluripotent stem cells” and published in Nature Biotechnology in 2014. As of April 16, 2023, this work has garnered 956 citations. The corresponding author of both the second and third most-cited works was Taylor R. In the realm of authorship, Taylor R’s contributions have amassed the most citations (1554 times). Within the top 10 articles, entries 1, 6, 7, 8, and 10 pivoted around foundational animal experiments, with articles 1, 6, 7, and 10 emphasizing transplantation therapies (stem cells/adipose tissue/islets) targeting diabetes remission. The residual articles were clinical investigations probing the effects and underlying mechanisms of diverse strategies (metabolic surgery and lifestyle interventions) envisioned for diabetes remission.

**Table 6:** Top 10 cited articles.

Rank	Title	Journal	Corresponding author	Year	citations
1	Reversal of diabetes with insulin-producing cells derived *in vitro* from human pluripotent stem cells (32)	*Nature* *Biotechnology*	Kieffer TJ	2014	956
2	Primary care-led weight management for remission of type 2 diabetes (DiRECT): an open-label, cluster-randomised trial (33)	*Lancet*	Taylor R	2018	842
3	Reversal of type 2 diabetes: normalisation of beta cell function in association with decreased pancreas and liver triacylglycerol (34)	*Diabetologia*	Taylor R	2011	712
4	Association of bariatric surgery with long-term remission of type 2 diabetes and with microvascular and macrovascular complications (35)	*JAMA*	Sjostrom L	2014	665
5	Reversal of nonalcoholic hepatic steatosis, hepatic insulin resistance, and hyperglycemia by moderate weight reduction in patients with type 2 diabetes (36)	*Diabetes*	Petersen KF	2005	627
6	Reversal of insulin-dependent diabetes using islets generated *in vitro* from pancreatic stem cells (37)	*Nature Medicine*	Peck AB	2000	551
7	Surgical implantation of adipose tissue reverses diabetes in lipoatrophic mice (38)	*Journal of * *Clinical * *Investigation*	Reitman ML	2000	474
8	Fibroblast growth factor 19 increases metabolic rate I and reverses dietary and leptlin-deficient diabetes (39)	*Endocrinology*	Stewart TA	2004	430
9	Association of an intensive lifestyle intervention with remission of type 2 diabetes (40)	*JAMA*	Gregg EW	2012	418
10	Prolonged diabetes reversal after intraportal xenotransplantation of wild-type porcine islets in immunosuppressed nonhuman primates (41)	*Nature Medicine*	Hering BJ	2006	405

### 3.2 Analysis of keywords

#### 3.2.1 High-frequency keywords analysis

We identified 606 keywords, with 34 exhibiting a frequency ≥30 (**Table 7**). The predominant five keywords were “bariatric surgery”, “mellitus”, “weight loss”, “type 2 diabetes”, and “beta cell function”. There are 15 salient keywords with centrality ≥0.1 (**Table 8**), and those with the highest centrality values are “beta cell”, “activation”, “beta cell function”, “adipose tissue”, and “clinical remission”.

**Table 7:** High frequency keywords list (frequency ≥30).

Rank	Keyword	Frequency	Centrality
1	bariatric surgery	266	0.09
2	mellitus	228	0.06
3	weight loss	166	0.11
4	type 2 diabete	161	0.04
5	beta cell function	123	0.24
6	metabolic surgery	89	0.02
7	insulin resistance	85	0.08
8	roux‐en‐y gastric bypass	83	0.03
9	sleeve gastrectomy	83	0.01
10	gastric bypass	83	0.01
11	diabetes remission	77	0.04
12	obesity	77	0.06
13	type 2 diabetes mellitus	76	0.02
14	therapy	74	0.09
15	glucose	71	0.05
16	association	71	0.01
17	obese patient	64	0.03
18	type 1 diabete	63	0.04
19	glycemic control	62	0.11
20	risk	55	0
21	mechanism	55	0.04
22	expression	54	0.09
23	diabetes mellitus	52	0.03
24	outcome	46	0
25	medical therapy	46	0.02
26	gastric bypass surgery	44	0.01
27	insulin sensitivity	44	0.01
28	life style intervention	37	0.01
29	oxidative stress	35	0.09
30	beta cell	35	0.25
31	body mass index	34	0.03
32	children	33	0.09
33	insulin	31	0.01
34	secretion	30	0

**Table 8:** High centrality keywords list (centrality ≥ 0.1).

Rank	Keyword	Centrality	Frequency
1	beta cell	0.25	35
2	activation	0.25	25
3	beta cell function	0.24	123
4	adipose tissue	0.18	13
5	clinical remission	0.17	9
6	t cell	0.15	24
7	sensitivity	0.13	25
8	weight loss	0.11	166
9	glycemic control	0.11	62
10	disease	0.11	26
11	*in vivo*	0.11	22
12	autoimmunity	0.11	7
13	cell	0.1	21
14	progression	0.1	14
15	antibody	0.1	7

#### 3.2.2 High-frequency keywords co-occurrence analysis

**Figure 8:**
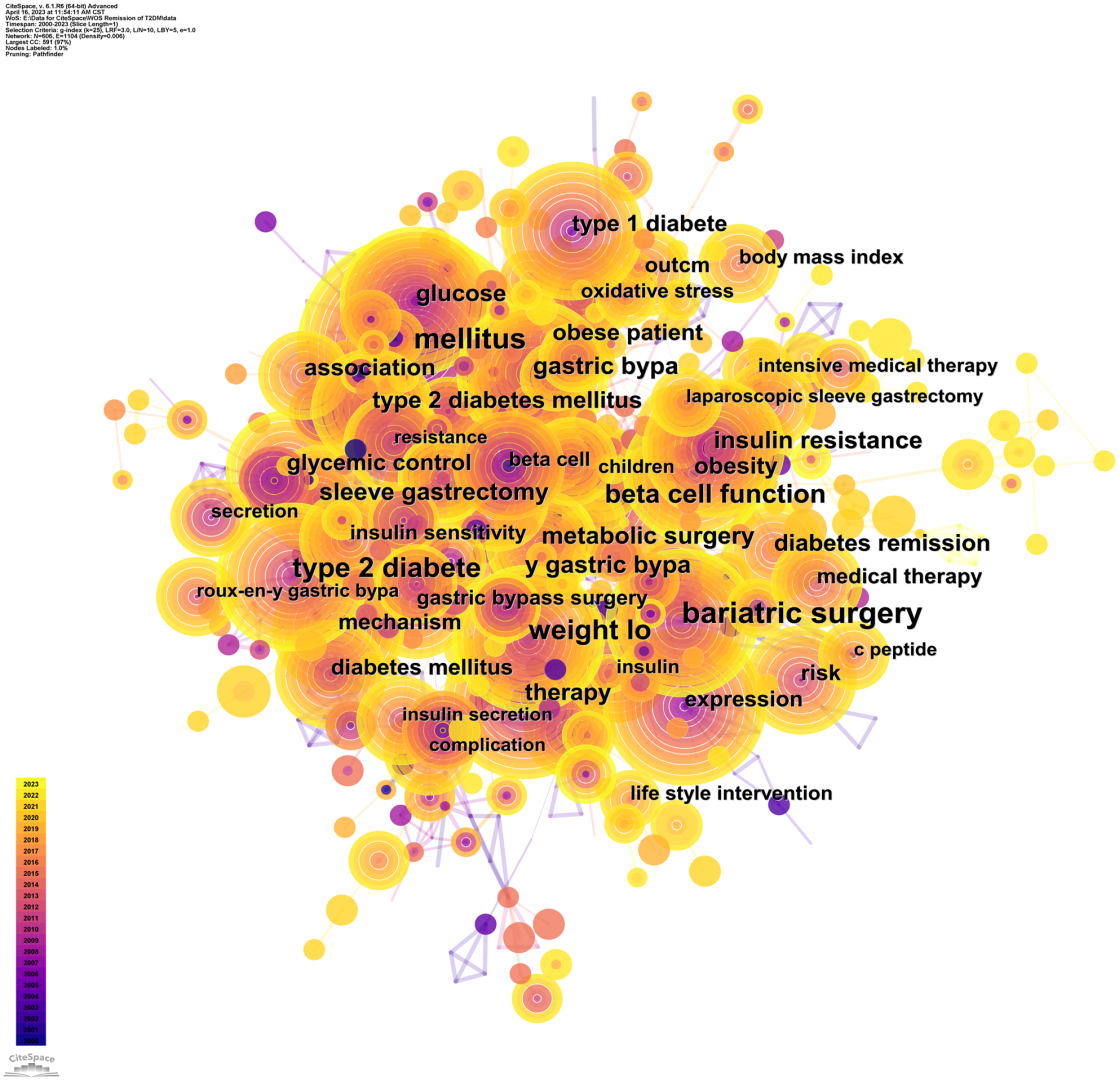
Keyword co-occurrence map. The larger the diameter of the circle, the higher the frequency, and the color from purple to yellow represents the year 2000 to 2023.

**Figure 8** renders a high-frequency keyword co-occurrence map. Essential keywords within this representation encompass “type 2 diabetes”, “type 1 diabetes”, “mellitus”, “glucose”, “therapy”, “obesity”, “weight loss”, “bariatric surgery”, “beta cells”, “beta cell function”, “insulin resistance”, and “expression”. Evidently, inquiries into diabetes remission predominantly concentrate on the nexus between obesity and the onset, progression, and remission of diabetes and the elucidation of remission via bariatric surgery and alternative weight loss modalities. Inquiries into remission mechanisms predominantly hone in on beta cell function and insulin resistance.

#### 3.2.3 High-frequency keywords emergence analysis

**Figure 9:**
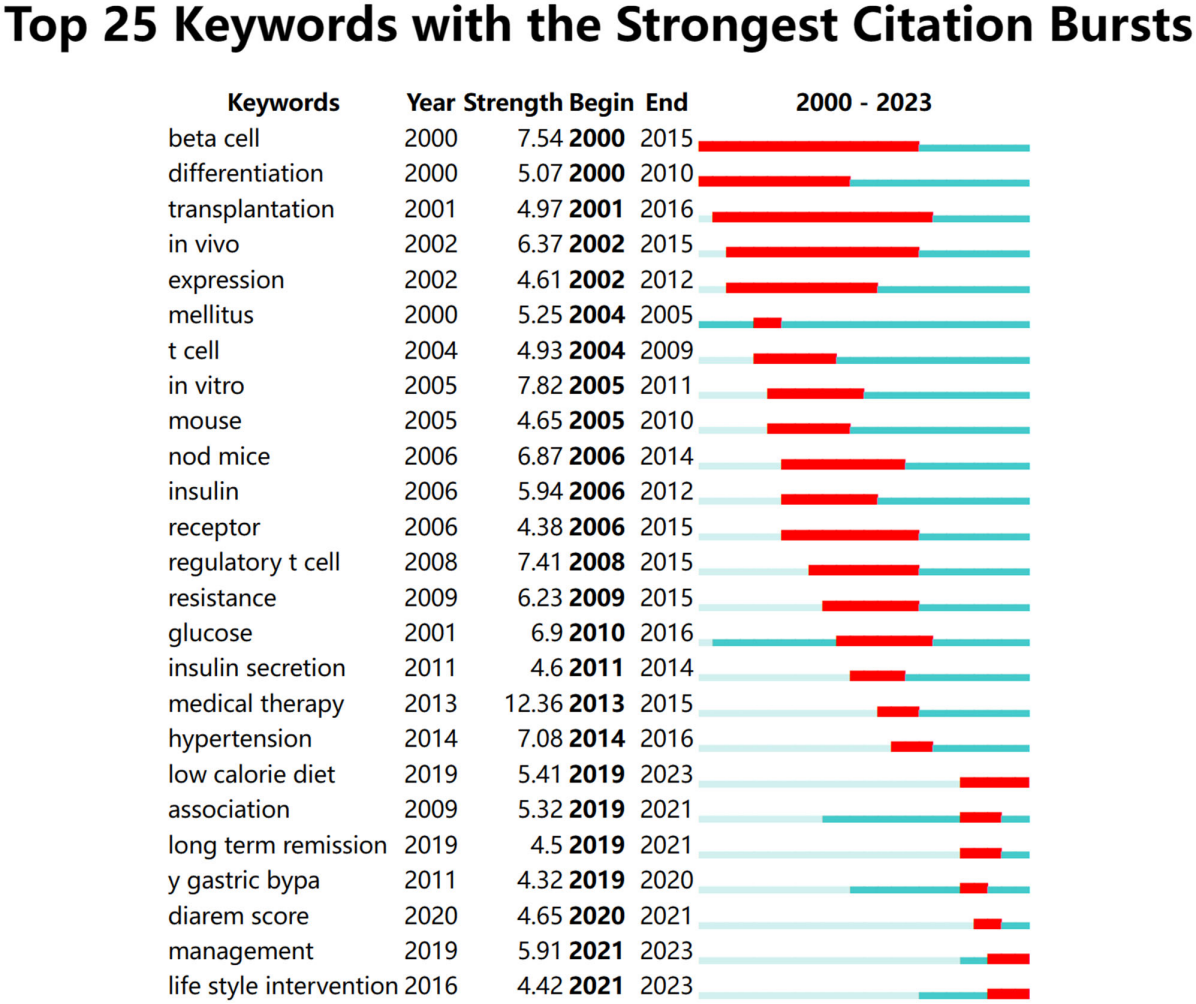
Keyword emergence map. The blue line represents the period from 2000 to 2023 and the red line represents the burst’s maintenance period.

We fabricated a map predicated on the intensity and chronology of keyword appearances (**Figure 9**), where “Strength” symbolizes the intensity of the sudden emergence. The higher the strength metric, the more profound its influence. “Begin” and “End” respectively signify the inception and culmination years of keyword emergence. It’s apparent that with the passage of time, research focal points transitioned from foundational experiments to clinical evaluations of diabetes remission, steered by bariatric surgery, lifestyle intervention protocols, and more. The prominence of “low-calorie diet”, “management”, and “lifestyle interventions” will persist through 2023, indicative of the current and forthcoming research thrusts in diabetes remission emphasizing lifestyle interventions and remission anchored in dietary therapy.

#### 3.2.4 Keyword cluster analysis

**Figure 10:**
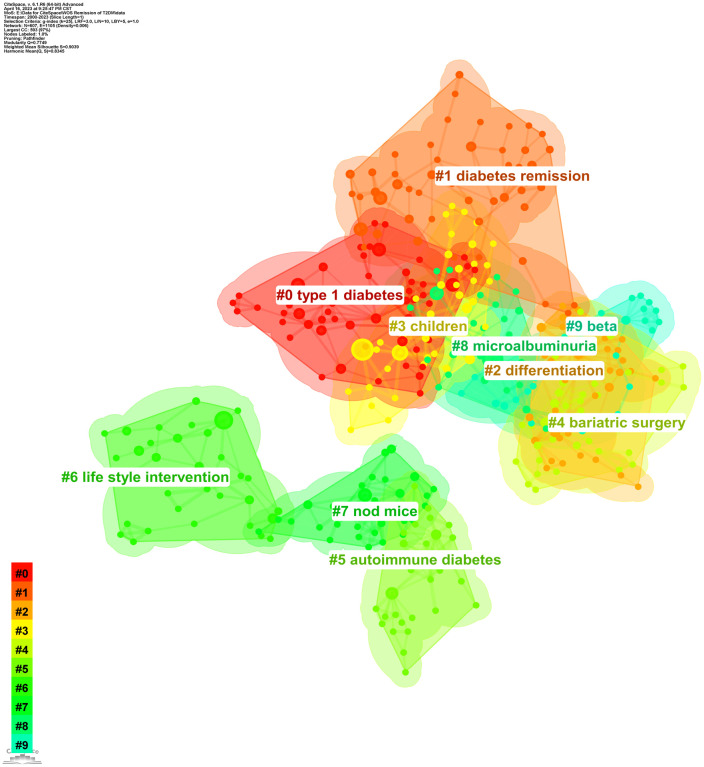
Keyword clustering map. Different color areas represent different clusters. The color gradation from 0 to 10 corresponds to a chronological progression from 2000 to 2023.

Hierarchical analysis was executed on the keywords, and the ten most significant clusters were chosen, resulting in **Figure 10**. A color gradation from 0 to 10 corresponds to a chronological progression from 2000 to 2023. The clustering labels encompassed #0 “type 1 diabetes”, #1 “diabetes remission”, #2 “differentiation”, #3 “children”, #4 “bariatric surgery”, #5 “autoimmune diabetes”, #6 “lifestyle intervention”, #7 “nod mice”, #8 “microalbuminuria”, and #9 “beta”. Intriguingly, labels 0, 1, 2, 3, 4, 8, and 9 exhibited partial overlap, amalgamating into a substantial conglomerate. This indicates that studies pertaining to these clustered themes manifest interconnectedness to varying extents. Conversely, labels 5, 6, and 7 delineate a comparatively autonomous entity, representative of emergent research trajectories in recent years.

The specifics of keyword clustering are elucidated in **Table 9**. An expansive cluster size implies a more considerable membership. The silhouette value acts as a metric of cluster member homogeneity; a larger value signifies greater member similarity. The silhouette values for each cluster, all surpassing 0.8, evince commendable cluster uniformity and consistency. Moreover, the table delineates the quartet of paramount keywords, in juxtaposition to label words. For instance, the most prominent cluster is denoted by label 1, termed “diabetes remission”. This label encapsulates quintessential keywords: diabetes remission, bariatric surgery, laparoscopic sleeve gastrectomy, remission criteria, and ABCD score. This underscores that research tethered to diabetes remission is most pervasive, with an emphasis on remission criteria, bariatric surgical interventions, and predictive models affiliated with diverse surgical procedures within the scope of diabetes remission.

**Table 9:** Keyword clustering information table.

ID	Size	Silhouette	Cluster	Top 5 keywords of the cluster based on LLR algorithm (loglikelihoodratio, p-level)
1	46	0.871	diabetes remission	diabetes remission (60.37, 0.0001); bariatric surgery (22.34,.0001); laparoscopic sleeve gastrectomy (19.61,0.0001); remission criteria(16.07,0.0001); ABCD score(16.03,0.0001)
0	43	0.871	type 1 diabete	type 1 diabetes (18.22, 0.0001); type 2 diabetes mellitus (14.16, 0.001); insulin secretion (11.59, 0.001); adipose tissue (9.24, 0.005); glucose metabolism (9.06, 0.005)
2	36	0.93	differentiation	differentiation (22.69, 0.0001); proliferation (17.73, 0.0001); hyperglycemia (17.12,0.0001); bariatric surgery (15.67, 0.0001); xenograft (12.12,0.001)
3	35	0.891	children	children (20,0.0001); beta cell function (18.14,0.0001); type 1 diabetes (15.53, 0.0001); c-peptide (12.93, 0.001); adolescent (12.18,0.001)
4	31	0.895	bariatric surgery	bariatric surgery (14.86, 0.001); arterial blood pressure (14.08, 0.001); angiotensinconverting enzyme (14.08, 0.001); mesenchymal stem cells (14.08, 0.001); engraftment (14.08,0.001)
8	31	0.912	microalbuminuria	microalbuminuria (18.96, 0.0001); resistance (17.35, 0.0001); mellitus (15.62, 0.0001);regression (14.21,0.001); association(11.48,0.001)
6	29	0.925	life style intervention	life style intervention (15.78, 0.0001); follow up (12.75, 0.001); quality of life (10.01, 0.005); reversibility (10.01, 0.005); prevention (9.95,0.005)
5	28	0.951	autoimmune diabetes	autoimmune diabetes (21.59, 0.0001); activation (17.15, 0.0001); phosphorylation (14.38, 0.001); abl tyrosine kinase (14.38, 0.001); c abl(14.38,0.001)
9	27	0.873	beta	beta (16.46, 0.0001); anti cd3 (16.46, 0.0001); islet neogenesis (12.67, 0.001); islets (9.83, 0.005); monoclonal antibody (8.95,0.005)
7	26	0.9	nod mice	nod mice (18.63, 0.0001); transplantation (16.35, 0.0001); therapy (14.35, 0.001); response (13.38, 0.001); diabetes remission (12.14,0.001)

#### 3.2.5 Keyword time zone map analysis

**Figure 11:**
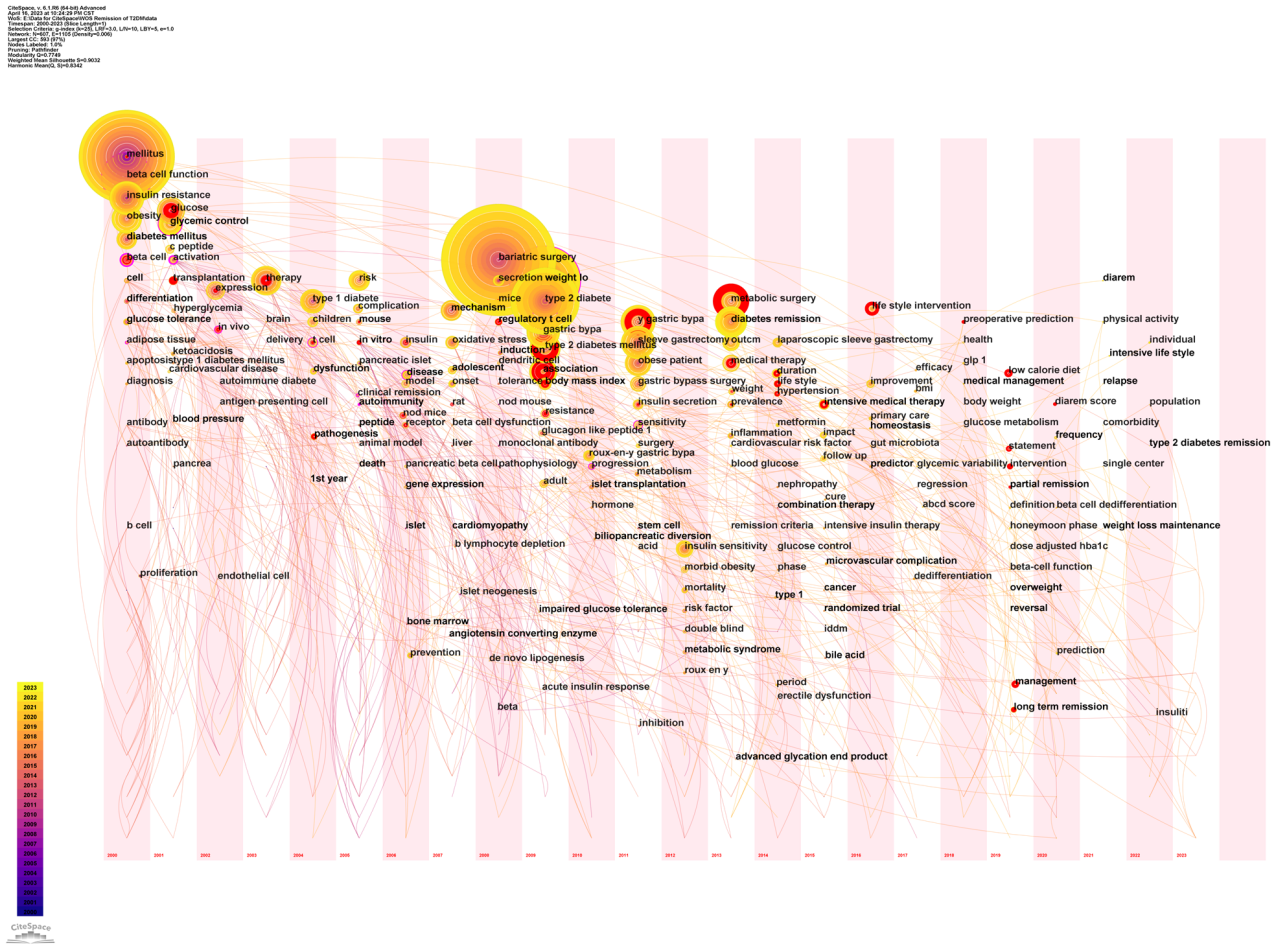
Keyword time zone map. The larger the diameter of the circle, the higher the frequency, and the color from purple to yellow represents the year 2000 to 2023.

Utilizing time as the ordinate, we envisaged temporal nodes and durations associated with each keyword, culminating in a keyword time-zone map (**Figure 11**). As delineated, a multitude of high-frequency, enduring keywords emerged in 2000, examples being “mellitus”, “beta cell function”, “insulin resistance”, and “obesity”. Spanning 2008 to 2013, a myriad of high-frequency, enduring keywords made their presence felt, including “bariatric surgery”, “weight loss”, “type 2 diabetes”, and “diabetes remission”.

#### 3.2.6 Keyword clustering timeline map analysis

**Figure 12:**
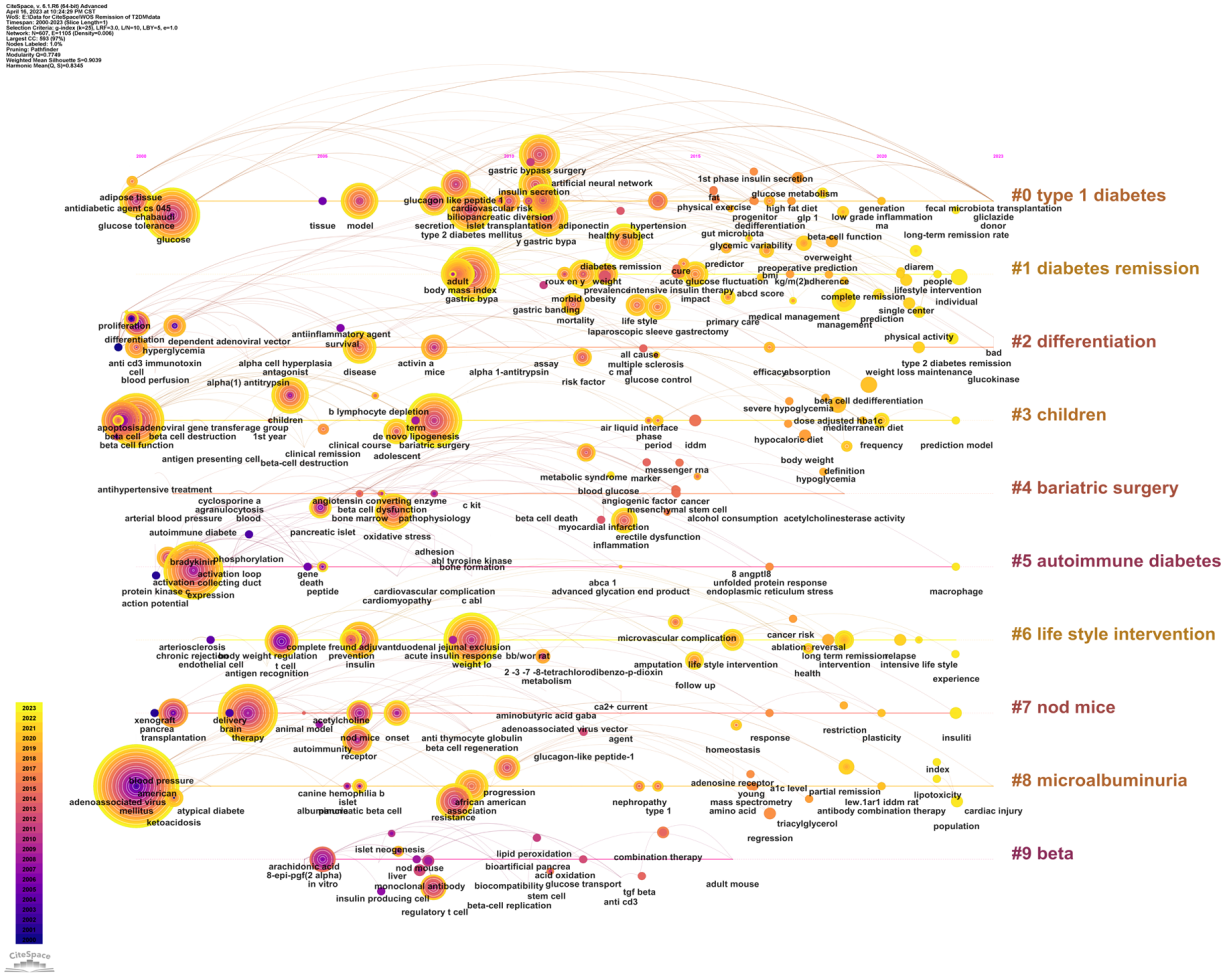
keyword clustering timeline map. The larger the diameter of the circle, the higher the frequency, and the color from purple to yellow represents the year 2000 to 2023.

With time as the ordinate, encapsulating temporal nodes and durations of each clustering label and all affiliated keywords, we procured a keyword-clustering timeline map (**Figure 12**). Analyzing the temporal trajectory of clustering, labels 0 “type 1 diabetes”, 2 “differentiation”, and 8 “microalbuminuria” enveloped the entire data acquisition span. However, barring “type 1 diabetes”, the keyword distribution in the other clusters was sporadic. Notwithstanding the succinct timespan of label 1 “diabetes remission”, the visualization illustrates that the keywords in this cluster are dense. Moreover, there exists an intertwined correlation with cluster 0 “type 1 diabetes”, marking it as a focal point of recent research endeavors.

## 4 Discussion

### 4.1 Analysis of articles publication

Through the analysis of article sources, one gains insights into the contemporary professional and preeminent teams and scholars in the realm of diabetes remission research. This elucidation offers directional guidance and foundational underpinning for associated investigators. Future trajectories of specialized domains can be inferred by an integrative assessment of publication tendencies amongst authors, institutions, nations, and journals.

This investigation revealed that, from an authorial standpoint, Taylor R boasts the highest quantity of both authored and cited manuscripts. Furthermore, his affiliated institution, the University of Newcastle, predominates in terms of manuscript publications. In the institutional collaboration network diagram (**Figure 3A**), the University of Newcastle predominantly liaised with the University of Glasgow in England. Within the authorial collaboration schematic (**Figure 4**), Taylor R emerged as the most dynamic, forging alliances with an array of teams and individuals, suggesting that he has rendered seminal contributions to the discipline of diabetes remission. Professor Roy Taylor stands as a luminary in the T2DM landscape, dedicating decades to the exploration of efficacious strategies for diabetes remission. He postulated that obesity is intricately intertwined with the genesis, evolution, and remission of diabetes. Through efficacious weight reduction, remission can be achieved, leading him to advocate the mechanism of diabetes progression and remission as the “twin vicious cycle theory” (42). His consortium executed a plethora of investigations into the attainment of diabetes remission via dietary curtailment, encompassing the “COUNTERPOINT study, COUNTERBALANCE study, and DiRECT study” (16). The quintessential exploration within Taylor’s oeuvre is the DiRECT study (43), which discerned that relying solely on dietary curtailment, the remission rates for T2DM patients at the 1- and 2-year marks stand at 45.6% and 35.6% respectively. Moreover, when these patients experienced a weight reduction exceeding 15 kg, the remission rates for T2DM at 1 and 2 years escalated to 86.1% and 70.0% respectively. Through the aforementioned series of investigations, Taylor also validated the “double vicious circle theory”. By shedding excess weight, patients can diminish superfluous fat burdens, curtail bodily fat proportions below the “personal fat threshold,” ameliorate liver and pancreatic fat accumulations, thereby enhancing insulin sensitivity. This facilitates the transformation of de-differentiated islet cells back into insulin-secreting β cells, culminating in T2DM remission. The findings from Professor Taylor’s team serve as a watershed in diabetes remission research, offering both a referential framework and directional compass for scholars in analogous domains.

America boasts the predominant count of published articles (**Table 1**). Numerous institutions within America maintain profound interactions, and among the top ten institutions in terms of article publication volume, five hail from America, collectively representing half of the overall article count published by these leading institutions (**Table 2**). Fifty percent of the preeminent ten journals that disseminate the majority of articles on diabetes remission are American entities (**Table 5**). In summation, academic establishments and researchers in America exhibit pronounced zeal for diabetes remission investigations, and intranational affiliations manifest both frequent and intimate collaborations. Publishing bodies in America also confer substantial emphasis on the advancement of diabetes remission. Consequently, America is poised to proffer augmented contributions to the diabetes remission domain in forthcoming years. A multitude of American scholars harbor an inclination towards metabolic surgery research to actualize diabetes remission, exemplified by teams such as Laferrère B’s ensemble at Columbia University (44), Schauer PR’s collective at the Cleveland Clinic (45), and Cummings DE’s contingent at Washington University (46), with evident interdisciplinary collaborations among these groups (**Figure 4**) (44). There also exist teams oriented towards dietary therapy for diabetes remission, such as the Look AHEAD Research Group (47). China ranks as the runner-up in terms of overall article publication (**Table 1**). A significant proportion of Chinese teams are engrossed in metabolic surgery research pertinent to diabetes remission, with exemplars like Zhu S’s faction from the Third Xiangya Hospital, Central South University (48), Jia W’s group from the Shanghai Jiao Tong University Affiliated Sixth People’s Hospital (49), and Zhang P’s assembly from the Shanghai Pudong Hospital of Fudan University (50). These teams also engage in intra-national collaborations (51). The most salient study on diabetes remission in England emanates from the series of investigations on dietary curtailment by Taylor R’s team, as elucidated previously. Moreover, certain English teams frequently establish collaborations with other European nations, notably Italy and Germany, with a predominant focus on metabolic surgery (52, 53). In Canada, certain teams, such as Lipscombe LL’s consortium from the University of Chicago (54) and the Carpentier AC’s coalition from the Centre de recherche du Centre hospitalier universitaire de Sherbrooke (55), delve into metabolic surgery, while others, such as Gerstein HC’s team, explore alternative remission strategies, encompassing hypoglycemic drugs and lifestyle interventions (56). Canada often forges collaborations with its southern neighbor, America (57). In Japan, metabolic surgery remains a paramount research area (58, 59). Spain hosts teams engrossed in metabolic surgery (60) and others probing the impact of dietary regimens on diabetes remission, exemplified by the CORDIOPREV study (61). In India, the majority of teams are engrossed in metabolic surgery (62, 63). Danish research related to diabetes remission is likewise dominated by metabolic surgery (64, 65).

International collaboration on diabetes remission is somewhat scant, primarily confined to geographically proximate nations. Broadly speaking, endeavors in diabetes remission necessitate an enhancement in collaborative synergies; however, there are understandable rationales behind the current state of affairs. One impeding factor is that prior to 2021, the definition of diabetes remission remained nebulous, and its criteria were discordant for an extended duration. Such ambiguities rendered the design and outcomes of pertinent studies variably heterogeneous, potentially curtailing intergroup collaborations. In August 2021, an assemblage of international multidisciplinary specialists, orchestrated by the American Diabetes Association, advocated for the term “remission” as the most apt descriptor. Furthermore, they posited that HbA1c <6.5%, ascertained a minimum of three months post the discontinuation of hypoglycemic therapeutic agents, should serve as the benchmark for remission (17). Given this resolution of terminological and criteria disputes concerning T2DM remission, it is anticipatory that ensuing research in diabetes remission will exhibit enhanced cohesion, fostering more streamlined alliances amongst research collectives and nations. Numerous modalities exist for diabetes remission, inclusive of bariatric surgery (14, 15), lifestyle interventions (16), short-term intensive insulin therapy (14), non-insulin hypoglycemic agents (66), immune modulation (67, 68), and transplantation (13, 69). Distinct research paradigms adhered to by various teams might circumscribe collaborative endeavors. Notwithstanding the inherent discrepancies in research foci and their consequent findings, there exists mutual edification and facilitation. Such interplay can be bolstered by the instigation of scholarly symposia and the fostering of scholarly exchanges. Additionally, the pandemic milieu of recent years has invariably impacted the momentum of pertinent clinical research and team interactions. Yet, during this health crisis, the publication trajectory concerning diabetes remission has persisted in its growth. With the ongoing stabilization of the epidemic, it is optimistically projected that investigations germane to diabetes remission will witness continued vigor, yielding more profound insights in the foreseeable future, and ultimately bestowing greater therapeutic dividends upon diabetic patients.

### 4.2 Analysis of research hotspots

In illuminating research focal points, we rendered various network diagrams predicated on keywords from the articles, presenting a comprehensive panorama of the specific content and temporal evolution of these focal points. The keyword with the preeminent frequency was “bariatric surgery”. Moreover, terms such as “weight loss”, “beta cell function”, and “insulin resistance”/”resistance” were prominent among high-frequency and high-centrality keywords. These terms, alongside their related keywords, made recurrent appearances in the maps elucidating keyword co-occurrence (**Figure 8**) and keyword clustering (**Figure 10**) in subsequent sections. This suggests a rich repository of studies centered on weight loss, particularly focusing on the remission of diabetes (primarily T2DM) via bariatric surgery. Investigations into remission mechanisms predominantly hinge on two cardinal pathogenic mechanisms: beta cell functionality and insulin resistance. Overweight/obesity-induced fatty liver and pancreas, which result from energy surfeit, are intricately linked to the pathogenesis and progression of T2DM (70, 71). Taylor’s proposition of the “twin cycle hypothesis” posits that excessive caloric intake culminates in significant adipose accumulation in the body, engendering insulin resistance and perturbations in beta cell functionality, eventually manifesting as T2DM. If the aforementioned sequela is attenuated via weight reduction, the natural corollary is the remission of diabetes (34, 42). An escalating corpus of literature underscores weight reduction as a pivotal factor in the remission of diabetes (33, 72–74). Currently, the two preeminent modalities for weight loss in the therapeutic regimen for T2DM encompass bariatric surgery and lifestyle modifications. These strategies have garnered significant attention in recent diabetes remission research, a trend corroborated in our subsequent keyword emergence (**Figure 9**) and keyword time-zone map (**Figure 11**) analysis.

Emergent terms, being high-frequency words discerned via fluctuations in keyword frequency within a delineated timeframe, encapsulate the zeitgeist of research during that epoch. The research was trifurcated based on the commencement and culmination of keyword emergence (**Figure 9**). The inaugural stage spanned 2000 to 2005, with research keywords predominantly revolving around “beta cells”, “differentiation”, “translation”, “*in vivo*”, “expression”, and “t-cell”. During this phase, a majority of studies embarked on exploratory endeavors into the feasibility and underlying mechanisms of remission [mainly Type 1 Diabetes Mellitus (T1DM)] in the context of transplantation-related or *in vivo* studies. They emphasized the pivotal role of transformations in beta cell functionality during the remission trajectory.

During this time frame, the primary emphasis of research in transplantation therapy was directed towards pancreas transplantation and islet transplantation. The pancreas could be transplanted either independently or in conjunction with the kidneys. In December 1966, a significant milestone was achieved when four surgeons, namely Kelly, Lillehei, Merkel, and Idezuk, hailing from the University of Minnesota Hospital in America, successfully conducted the inaugural pancreas kidney transplantation procedure for two individuals with diabetes and renal failure (75). This groundbreaking achievement marked the commencement of a novel epoch in the realm of clinical pancreas transplantation, offering hope to individuals afflicted with diabetes and renal failure, who were facing imminent mortality. Subsequently, a substantial volume of pancreas transplantation procedures has been conducted globally, resulting in a notable rise in the post-transplantation insulin detachment rate from 40% to exceeding 70% within the preceding decade (76).

Islet transplantation, which had its genesis in the mid-1970s, resurged in prominence due to the successful execution of the Edmonton protocol in 2000 (77). Edmond administered pancreatic islet transplantation to 7 T1DM patients presenting with pronounced hypoglycemia and metabolic instabilities, culminating in all the patients ceasing insulin administration. The mean HbA1c values normalized, blood sugar variability markedly diminished, and no instances of severe hypoglycemia were reported subsequently. Absence of noteworthy contraindications or adverse reactions, and the obviation of hormonal therapy were also notable. In 2022, the Edmonton protocol team published findings from a cohort study conducted at a single center, examining the outcomes of islet transplantation up to 20 years post-transmission (78). The study encompassed a median follow-up period of 7.4 years and included 255 patients who underwent allogeneic islet transplantation. Of these patients, 78 (70%) experienced sustained graft survival, while 77 (30%) experienced non-sustained graft survival. Among the total patient population, 201 individuals (79%) achieved insulin independence. Notably, the rate of insulin independence was higher among transplant survivors compared to non-transplant survivors. The prevalence and stability of HbA1c levels and fasting blood glucose levels, along with a notable improvement in hypoglycemia, were observed over the course of the follow-up period. These findings provide evidence for the safety and efficacy of islet transplantation as a treatment modality for T1DM. Furthermore, the continued advancement and implementation of relevant technologies in the field of islet transplantation hold promise for achieving a definitive cure for T1DM in the future.

Simultaneously, the Edmonton protocol team conducted a comparative analysis of the follow-up data pertaining to patients who underwent pancreatic islet transplantation (n=266) and pancreas transplantation pancreas transplantation (n=146) at the research center within the same time frame (79). The findings revealed that while the pancreas transplantation group exhibited elevated mortality rates, surgery-related complications, and readmission rates, it demonstrated superior outcomes in terms of insulin independence, graft survival rate, and blood glucose control. These results suggest that pancreas transplantation holds greater potential than islet transplantation in diabetes remission.

Since the year 2000, numerous investigations have been conducted in the domains of islet transplantation and pancreas transplantation. Both approaches to transplantation offer potential benefits such as reducing patients’ dependence on insulin, mitigating the occurrence of severe hypoglycemic events, and enhancing the management of blood sugar levels. In the wake of Edmonton protocol, a plethora of clinical and animal studies were conducted, yielding favorable experimental outcomes (80, 81). Such pioneering results further galvanized preliminary investigations into immunosuppressive modalities, stem cell transplantation, and genetic therapeutic interventions (37, 82–84), and catalyzed a myriad of studies on beta cell function (85, 86). This era was marked by a profusion of studies investigating potential strategies for the reversal/remission of T1DM. In 2005, the American Diabetes Association (ADA) promulgated an article titled “Thirty years of investigating the autoimmune basis for type 1 diabetes: why can’t we prevent or reverse this disease?” (87). This manuscript offered a retrospective examination of the knowledge amassed during this time, and in tandem with the analysis of these research endeavors, proffered guidance for forthcoming inquiries with aspirations to translate these findings into efficacious modalities for the prevention or reversal of T1DM.

From 2005 to 2010, keywords such as “*in vitro*”, “mouse”, “nod mice”, “receptor”, and “regulatory T cells” manifested in succession, signifying that *in vitro* and mouse experiments transitioned into primary research foci. A significant portion of investigations during this timeframe endeavored to elucidate the phenomena and underlying mechanisms of diabetes remission via foundational experiments. This epoch, representing the second stage, extended until 2015. Concurrently, there was an intensified quest to identify modalities for diabetes reversal beyond mere transplantation. This pursuit was underscored in 2006 when D’Amour et al. disseminated a seminal article detailing the synthesis of endocrine pancreatic cells (88). These cells exhibit a secretion profile comprising insulin, glucagon, somatostatin, pancreatic polypeptides, and auxin-releasing peptides. This pioneering investigation marked the inaugural documentation of the derivation of hormone-expressing endocrine pancreatic cells from human embryonic stem cells subjected to *in vitro* differentiation. The research posits that the transplantation of insulin-producing pancreatic beta cells, with the intent to augment the depleting reserve of such cells in T1DM patients, could potentially herald a therapeutic paradigm or even a definitive resolution for the condition, thereby rejuvenating optimism for diabetes remission.

Subsequent to 2010, terms such as “medical therapy”, “glucose”, “hypertension”, and other clinical pertinent keywords emerged, denoting a transition in the research trajectory on diabetes remission from a foundational to a clinical orientation. Spanning 2010 to 2015, a plethora of clinical evaluations substantiated the premise that short-term intensive insulin therapy possesses the efficacy to facilitate T2DM remission (89, 90). This evolution in research emphasis signified a shift from foundational experiments towards clinical trials, with the primary research axis transitioning from T1DM to T2DM.

In 2016, coinciding with the promulgation of guidelines for metabolic surgery as an intervention for diabetes (91), the research interest in employing this modality for achieving T2DM remission experienced a notable upsurge. A considerable body of literature attested that metabolic surgery emerged as the technique boasting the superior remission rate amongst all extant remission strategies (14, 15). Consequently, the research vigor persisted, yielding substantive findings. Investigations concerning its remission mechanisms, prognostic indicators, and predictive models attained a high degree of sophistication (92, 93). The triumphant outcomes of metabolic surgery in facilitating T2DM remission illuminated the potential of weight reduction as a realistic avenue towards T2DM remission. This spurred researchers to delve deeper into alternative methodologies for weight reduction conducive to T2DM remission. Subsequent investigations corroborated that a substantial proportion of patients, nearing half, could attain a drug-free remission state for T2DM solely via dietary and lifestyle alterations aimed at weight loss (15, 16, 94). In the wake of these revelations, lifestyle intervention swiftly ascended as a pivotal research domain within the wider landscape of diabetes remission, succeeding metabolic surgery. Specifically, dietary regimens underscored by caloric restriction emerged as focal points. The 2021 remission guideline for T2DM accentuated the paramountcy of lifestyle interventions in ameliorating diabetes (17). In 2020 (95) and 2022 (96), ADA unveiled two distinct guidelines emphasizing dietary therapy for T2DM remission. This trajectory underscores the indispensable role of diet therapy in mitigating diabetes, heralding it as an epicenter of research endeavors both presently and in foreseeable future epochs. Post-2019, a preponderance of investigations has gravitated towards clinical research, characterized by keywords such as “low-calorie diet”, “long-term remission”, “roux‐en‐y gastric bypass”, “management”, and “lifestyle intervention”. This inclination elucidates that within the clinical domain of diabetes remission, bariatric surgery and lifestyle interventions underscored by a low-calorie diet remain the predominant foci. The triad of “low-calorie diet”, “management”, and “lifestyle intervention” are poised to maintain their prominence through 2023, signifying that contemporary and imminent research nuclei in diabetes remission revolve around lifestyle interventions fortified by dietary regimens. The manifestation of these keywords attests to the prevailing trend that research on diabetes remission through bariatric surgery and lifestyle interventions targeting weight reduction has gained momentum in recent years, and prognostications suggest an inclination towards lifestyle intervention-centric research in the near future. This trend is corroborated by observations from the keyword time zone map (**Figure 11**) and the keyword clustering timeline map (**Figure 12**).

Various strategies have been proposed to realize diabetes remission, encompassing metabolic surgery, lifestyle intervention, insulin intensive therapy, and non-insulin hypoglycemic drugs (14). Metabolic surgery is the most efficacious, with a maximum remission rate of approximately 70%–80% at 1–2 years after surgery (97, 98), followed by lifestyle interventions with caloric restriction, with a 40%–80% average remission rate at 1 year after intervention (95), and intensive insulin therapy, which can also provide remission in 30%–70% of patients with T2DM (66, 99). However, the efficacy of oral hypoglycemic drugs as an intervention for diabetes remission understudied, with limited research indicating remission rates predominantly below 30% (14, 100).

Lifestyle interventions commonly prioritize interventions involving low calorie or even very low calorie diet. However, patients often encounter difficulties in maintaining adherence due to intense and persistent hunger. In cases where patients are unable to sustain dietary control after restricting their intake, weight regain may occur, thereby impacting the attainment and sustenance of remission. Metabolic surgery is specifically designed for individuals with severe obesity, particularly those experiencing complications. Nevertheless, its application is limited in scope and it is an invasive treatment option that is often met with low patient acceptance. Nevertheless, there is a scarcity of research examining the efficacy of antidiabetic medications in remission of T2DM, despite their relatively high acceptance and compliance rates. Current research findings indicate that these drugs exhibit inferior remission outcomes compared to lifestyle intervention and metabolic surgery. However, in recent times, novel hypoglycemic drugs, such as glucagon-like peptide-1 receptor agonists (GLP-1RA), Sodium-glucose cotransporter-2 inhibitors (SGLT-2i), and dipeptidyl peptidase-4 inhibitors, have been introduced both domestically and internationally (101–103). These medications, which operate through diverse mechanisms, demonstrate favorable hypoglycemic effects and offer potential benefits in preserving or even safeguarding pancreas function. Among these, SGLT-2i, GLP-1RA, dual glucose-dependent diabetes peptides (GIP), and the GLP-1RA tirzepatide exhibit a range of additional advantages beyond glucose reduction, including potential mechanisms for remission of T2DM, such as weight loss (71, 103–105). Hence, there exist numerous unexplored and enhanced areas within the domain of diabetes remission research that warrant future investigation. These areas encompass evaluating the efficacy of recently introduced hypoglycemic medications, examining the potential benefits of combining diverse mitigation strategies to enhance compliance and response rates, and exploring the impact of improved care and management on increasing remission rates, among others.

Given that China has the highest number of diabetes patients globally, the imperative to discover effective approaches for treating and remission of diabetes is even more pronounced. Chinese traditional medicine possesses distinct advantages in the treatment of various diseases. While certain medications, such as Tianqi capsule, Jin qi jiang tang tablet, and Jin li da granule, have demonstrated efficacy in remission/reversal of prediabetes (106), limited research exists on their effectiveness in remission of clinical diabetes. Consequently, further investigation is warranted to explore mitigation strategies tailored to the Chinese population, with particular emphasis on harnessing the unique attributes of traditional Chinese medicine for the treatment and remission of diabetes.

## 5 Strengths and limitations

This study employed two bibliometric software packages to furnish a holistic multidimensional map display. Marking a pioneering endeavor, this study probed publications on diabetes remission, delineating their evolutionary trajectories in a lucid, unbiased, and precise manner, thus offering invaluable insights for clinicians and academicians in this domain. However, there exist certain constraints intrinsic to this study. Our chosen search strategy, anchored in title-based queries, might have inadvertently bypassed articles which, though not explicitly referencing “diabetes remission” in the title, incorporated pertinent discussions on the topic within the main body of the text. We engaged in iterative deliberations regarding the optimal search strategy and consequently instituted a subject-based search. Notwithstanding the identification of a plethora of articles via this approach, a preliminary analysis upon their integration into the software discerned the presence of numerous high-frequency keywords, keyword clusters, and other analytic components that bore tenuous or negligible relevance to diabetes remission. Given the potential that certain articles, perhaps those centered on bariatric surgery, might delve into facets specifically tied to the surgical procedure rather than glycemic control/remission, there was a palpable risk of introducing considerable analytical bias. Consequently, to circumvent such pitfalls, we prudently elected to constrain our search to titles alone.

## 6 Conclusion

In general, the study of diabetes remission is relatively popular at present. Currently, bariatric surgery and lifestyle interventions are widely studied strategies, particularly lifestyle interventions based on dietary therapy, which are becoming increasingly popular. We hope that this study will help researchers to better understand the overall trends in this field and provide guidance for future research.

## Data availability statement

The raw data supporting the conclusions of this article will be made available by the authors, without undue reservation.

## Author contributions

CL: Conceptualization, Methodology, Project administration, Supervision, Writing – review & editing. XY: Data curation, Formal Analysis, Methodology, Software, Visualization, Writing – original draft, Writing – review & editing. ZH: Data curation, Formal Analysis, Investigation, Methodology, Software, Visualization, Writing – review & editing. QC: Data curation, Formal Analysis, Investigation, Methodology, Software, Visualization, Writing – review & editing, Resources. YC: Conceptualization, Formal Analysis, Supervision, Writing – review & editing. GC: Conceptualization, Investigation, Methodology, Project administration, Supervision, Writing – review & editing.

## References

[1] Sidik SM. Diabetes and obesity are rising globally - but some nations are hit harder. Nature (2023) 1–5. doi: 10.1038/d41586-023-00676-z 36890314

[2] Chew NWS Ng CH Tan DJH Kong G Lin C Chin YH. The global burden of metabolic disease: data from 2000 to 2019. Cell Metab (2023) 35(3):414–28. doi: 10.1016/j.cmet.2023.02.003 36889281

[3] IDF diabetes atlas. 10th ed. International Diabetes Federation (2022). Available at: http://www.diabetesatlas.org/.

[4] Wang X Lin Y Xiong Y Zhang S He Y He Y. Using an optimized generative model to infer the progression of complications in type 2 diabetes patients. BMC Med Inform Decis Mak (2022) 22(1):174. doi: 10.1186/s12911-022-01915-5 35778708 PMC9250218

[5] Lim LL Chow E Chan JCN. Cardiorenal diseases in type 2 diabetes mellitus: clinical trials and real-world practice. Nat Rev Endocrinol (2023) 19(3):151–63. doi: 10.1038/s41574-022-00776-2 36446898

[6] Kuan V Denaxas S Patalay P Nitsch D Mathur R Gonzalez-Izquierdo A. Identifying and visualising multimorbidity and comorbidity patterns in patients in the English national health service: a population-based study. Lancet Digit Health (2023) 5(1):e16–27. doi: 10.1016/S2589-7500(22)00187-X 36460578

[7] Wang JM Liu QP Zhang ML Gong C Liu SD Chen WY. [Effectiveness of different screening strategies for type 2 diabete on preventing cardiovascular diseases in a community-based Chinese population using a decision-analytic Markov model]. Beijing Da Xue Xue Bao Yi Xue Ban (2022) 54(3):450–7. doi: 10.19723/j.issn.1671-167X.2022.03.009 PMC9197700 35701121

[8] Bao H. Intervention effect of mindfulness-based cognitive therapy on diabetes-related distress and self-care. Iran J Public Health (2022) 51(3):606–14. doi: 10.18502/ijph.v51i3.8937 PMC9276602 35865066

[9] Arsh A Afaq S Carswell C Bhatti MM Ullah I Siddiqi N. Effectiveness of physical activity in managing co-morbid depression in adults with type 2 diabetes mellitus: a systematic review and meta-analysis. J Affect Disord (2023) 329:448–59. doi: 10.1016/j.jad.2023.02.122 36868385

[10] John D Montvida O Paul SK. Racial disparity in the co-occurrence of depression and type 2 diabetes mellitus. An electronic medical record study involving African American and White Caucasian adults from the US. J Affect Disord (2023) 330:173–9. doi: 10.1016/j.jad.2023.02.097 36868390

[11] Wang L Peng W Zhao Z Zhang M Shi Z Song Z. Prevalence and treatment of diabetes in China, 2013-2018. JAMA (2021) 326(24):2498–506. doi: 10.1001/jama.2021.22208 PMC8715349 34962526

[12] Xie J Wang M Long Z Ning H Li J Cao Y. Global burden of type 2 diabetes in adolescents and young adults, 1990-2019: systematic analysis of the global burden of disease study 2019. BMJ (2022) 379:e072385. doi: 10.1136/bmj-2022-072385 36740855 PMC9727920

[13] Mudaliar S. The evolution of diabetes treatment through the ages: from starvation diets to insulin, incretins, SGLT2-inhibitors and beyond. J Indian Inst Sci (2023) 1(2023):1–11. doi: 10.1007/s41745-023-00357-w PMC9942084 36845885

[14] Kim J Kwon HS. Not control but conquest: strategies for the remission of type 2 diabetes mellitus. Diabetes Metab J (2022) 46(2):165–80. doi: 10.4093/dmj.2021.0377 PMC8987695 35385632

[15] Shibib L Al-Qaisi M Ahmed A Miras AD Nott D Pelling M. Reversal and remission of T2DM - an update for practitioners. Vasc Health Risk Manag (2022) 18:417–43. doi: 10.2147/VHRM.S345810 PMC9206440 35726218

[16] Salis S Anjana RM Unnikrishnan R Syed S Mohan V. Remission of type 2 diabetes: how, when, and for whom? J Assoc Physicians India (2022) 70(8):11–2. doi: 10.5005/japi-11001-0078 36082730

[17] Riddle MC Cefalu WT Evans PH Gerstein HC Nauck MA Oh WK. Consensus report: definition and interpretation of remission in type 2 diabetes. Diabetes Care (2021) 44(10):2438–44. doi: 10.2337/dci21-0034 PMC8929179 34462270

[18] Taylor R Barnes AC. Can type 2 diabetes be reversed and how can this best be achieved? James Lind Alliance research priority number one. Diabetes Med (2019) 36(3):308–15. doi: 10.1111/dme.13851 30378706

[19] Zhou W Bai X Yang Y Huang M Zheng Q Wu J. Revelations of delirium subtype research: a bibliometric analysis of publications in the past twenty years in the field. Asian J Psychiatr (2023) 83:103561. doi: 10.1016/j.ajp.2023.103561 36989982

[20] de-la-Fuente-Robles YM Ricoy-Cano AJ Albín-Rodríguez AP López-Ruiz JL Espinilla-Estévez M. Past, present and future of research on wearable technologies for healthcare: a bibliometric analysis using scopus. Sensors (Basel) (2022) 22(22):8599. doi: 10.3390/s22228599 36433195 PMC9696945

[21] Ding X Yang Z. Knowledge mapping of platform research: a visual analysis using VOSviewer and CiteSpace. Electron Commer Res (2020) 22(3):787–809. doi: 10.1007/s10660-020-09410-7

[22] Shen Z Ji W Yu S Cheng G Yuan Q Han Z. Mapping the knowledge of traffic collision reconstruction: a scientometric analysis in CiteSpace, VOSviewer, and SciMAT. Sci Justice (2023) 63(1):19–37. doi: 10.1016/j.scijus.2022.10.005 36631179

[23] Ran X Deng Y Uppuluri NST Li B Zheng Y Chen P. Hotspots and future trends of phosphorus recycling from livestock manure: a bibliometric review. Sci Total Environ (2023) 892:164346. doi: 10.1016/j.scitotenv.2023.164346 37236471

[24] Mohamed B Marzouk M. Bibliometric analysis and visualisation of heritage buildings preservation. Heritage Sci (2020) 11(1):1–20. doi: 10.1186/s40494-023-00947-y

[25] Wu H Sun Z Tong L Wang Y Yan H Sun Z. Bibliometric analysis of global research trends on male osteoporosis: a neglected field deserves more attention. Arch Osteoporos (2021) 16(1):154. doi: 10.1007/s11657-021-01016-2 34632530

[26] Luo Y Wang T Chen Z Zhang G. Knowledge domain and emerging trends in beta-cell research: A bibliometric and knowledge-map analysis. Front Endocrinol (Lausanne) (2023) 13:1086667. doi: 10.3389/fendo.2022.1086667 36743933 PMC9892706

[27] Xu C Jiang R Liu JY. Emerging trends and hot spots in subacute thyroiditis research from 2001 to 2022: a bibliometric analysis. Front Endocrinol (Lausanne) (2023) 14:1144465. doi: 10.3389/fendo.2023.1144465 37008914 PMC10064097

[28] Zhang L Zhang H Xie Q Xiong S Jin F Zhou F. A bibliometric study of global trends in diabetes and gut flora research from 2011 to 2021. Front Endocrinol (Lausanne) (2022) 13:990133. doi: 10.3389/fendo.2022.990133 36339425 PMC9633665

[29] Yuan N Wang L Li Z Zhang X. Thyroid diseases during pregnancy: bibliometric analysis of scientific publications. Endocr Metab Immune Disord Drug Targets (2022) 22(2):247–58. doi: 10.2174/1871530321666210203214142 33538681

[30] Li F Xie W Han Y Li Z Xiao J. Bibliometric and visualized analysis of exercise and osteoporosis from 2002 to 2021. Front Med (Lausanne) (2022) 9:944444. doi: 10.3389/fmed.2022.944444 36569140 PMC9773261

[31] Markscheffel B Schrter F. Comparison of two science mapping tools based on software technical evaluation and bibliometric case studies. COLLNET J Scientometr Inf Manag (2021) 15(2):365–96. doi: 10.1080/09737766.2021.1960220

[32] Rezania A Bruin JE Arora P Rubin A Batushansky I Asadi A. Reversal of diabetes with insulin-producing cells derived *in vitro* from human pluripotent stem cells. Nat Biotechnol (2014) 32(11):1121–33. doi: 10.1038/nbt.3033 25211370

[33] Lean ME Leslie WS Barnes AC Brosnahan N Thom G McCombie L. Primary care-led weight management for remission of type 2 diabetes (DiRECT): an open-label, cluster-randomised trial. Lancet (2018) 391(10120):541–51. doi: 10.1016/S0140-6736(17)33102-1 29221645

[34] Lim EL Hollingsworth KG Aribisala BS Chen MJ Mathers JC Taylor R. Reversal of type 2 diabetes: normalisation of beta cell function in association with decreased pancreas and liver triacylglycerol. Diabetologia (2011) 54(10):2506–14. doi: 10.1007/s00125-011-2204-7 PMC3168743 21656330

[35] Sjöström L Peltonen M Jacobson P Ahlin S Andersson-Assarsson J Anveden Å. Association of bariatric surgery with long-term remission of type 2 diabetes and with microvascular and macrovascular complications. JAMA (2014) 311(22):2297–304. doi: 10.1001/jama.2014.5988 24915261

[36] Petersen KF Dufour S Befroy D Lehrke M Hendler RE Shulman GI. Reversal of nonalcoholic hepatic steatosis, hepatic insulin resistance, and hyperglycemia by moderate weight reduction in patients with type 2 diabetes. Diabetes (2005) 54(3):603–8. doi: 10.2337/diabetes.54.3.603 PMC2995496 15734833

[37] Ramiya VK Maraist M Arfors KE Schatz DA Peck AB Cornelius JG. Reversal of insulin-dependent diabetes using islets generated *in vitro* from pancreatic stem cells. Nat Med (2000) 6(3):278–82. doi: 10.1038/73128 10700229

[38] Gavrilova O Marcus-Samuels B Graham D Kim JK Shulman GI Castle AL. Surgical implantation of adipose tissue reverses diabetes in lipoatrophic mice. J Clin Invest (2000) 105(3):271–8. doi: 10.1172/JCI7901 PMC377444 10675352

[39] Fu L John LM Adams SH Yu XX Tomlinson E Renz M. Fibroblast growth factor 19 increases metabolic rate and reverses dietary and leptin-deficient diabetes. Endocrinology (2004) 145(6):2594–603. doi: 10.1210/en.2003-1671 14976145

[40] Gregg EW Chen H Wagenknecht LE Clark JM Delahanty LM Bantle J. Association of an intensive lifestyle intervention with remission of type 2 diabetes. JAMA (2012) 308(23):2489–96. doi: 10.1001/jama.2012.67929 PMC4771522 23288372

[41] Hering BJ Wijkstrom M Graham ML Hårdstedt M Aasheim TC Jie T. Prolonged diabetes reversal after intraportal xenotransplantation of wild-type porcine islets in immunosuppressed nonhuman primates. Nat Med (2006) 12(3):301–3. doi: 10.1038/nm1369 16491083

[42] Taylor R Al-Mrabeh A Sattar N. Understanding the mechanisms of reversal of type 2 diabetes. Lancet Diabetes Endocrinol (2019) 7(9):726–36. doi: 10.1016/S2213-8587(19)30076-2 31097391

[43] Lean MEJ Leslie WS Barnes AC Brosnahan N Thom G McCombie L. Durability of a primary care-led weight-management intervention for remission of type 2 diabetes: 2-year results of the DiRECT open-label, cluster-randomised trial. Lancet Diabetes Endocrinol (2019) 7:344–55. doi: 10.1016/S2213-8587(19)30068-3 30852132

[44] Ligon C Shah A Prasad M Laferrère B. Preintervention clinical determinants and measured β-cell function as predictors of type 2 diabetes remission after Roux-en-Y gastric bypass surgery. Diabetes Care (2021) 44(10):2427–34. doi: 10.2337/dc21-0395 PMC8929185 34400479

[45] Kirwan JP Courcoulas AP Cummings DE Goldfine AB Kashyap SR Simonson DC. Diabetes remission in the alliance of randomized trials of medicine versus metabolic surgery in type 2 diabetes (ARMMS-T2D). Diabetes Care (2022) 45(7):1574–83. doi: 10.2337/dc21-2441 PMC9490448 35320365

[46] Cohen RV Pinheiro JC Schiavon CA Salles JE Wajchenberg BL Cummings DE. Effects of gastric bypass surgery in patients with type 2 diabetes and only mild obesity. Diabetes Care (2012) 35(7):1420–8. doi: 10.2337/dc11-2289 PMC3379595 22723580

[47] Look AHEAD Research Group. Effects of a long-term lifestyle modification programme on peripheral neuropathy in overweight or obese adults with type 2 diabetes: the Look AHEAD study. Diabetologia (2017) 60(6):980–8. doi: 10.1007/s00125-017-4253-z PMC5423967 28349174

[48] Luo P Cao Y Li P Wang G Song Z Li W. Insulin resistance remission following laparoscopic Roux-en-Y gastric bypass and laparoscopic sleeve gastrectomy in Chinese type 2 diabetes mellitus patients with a body mass index of 27.5-32.5 kg/m2. Front Physiol (2021) 12:772577. doi: 10.3389/fphys.2021.772577 34819878 PMC8606571

[49] Li S Yu H Zhang P Tu Y Xiao Y Yang D. The nonlinear relationship between psoas cross-sectional area and BMI: a new observation and its insights into diabetes remission after Roux-en-Y gastric bypass. Diabetes Care (2021) 44(12):2783–6. doi: 10.2337/dc20-2907 PMC8669530 34645667

[50] Wang T Shen Y Qiao Z Wang Y Zhang P Yu B. Comparison of diabetes remission and micronutrient deficiency in a mildly obese diabetic rat model undergoing SADI-S versus RYGB. Obes Surg (2019) 29(4):1174–84. doi: 10.1007/s11695-018-03630-5 30610678

[51] Li M Liu Y Lee WJ Shikora SA Robert M Wang W. Efficacy and safety of one anastomosis gastric bypass versus Roux-en-Y gastric bypass for type 2 diabetes remission (ORDER): protocol of a multicentre, randomised controlled, open-label, superiority trial. BMJ Open (2022) 12(9):e062206. doi: 10.1136/bmjopen-2022-062206 PMC9528602 36175102

[52] Mingrone G Panunzi S De Gaetano A Guidone C Iaconelli A Capristo E. Metabolic surgery versus conventional medical therapy in patients with type 2 diabetes: 10-year follow-up of an open-label, single-centre, randomised controlled trial. Lancet (2021) 397(10271):293–304. doi: 10.1016/S0140-6736(20)32649-0 33485454

[53] Mingrone G Panunzi S De Gaetano A Guidone C Iaconelli A Nanni G. Bariatric-metabolic surgery versus conventional medical treatment in obese patients with type 2 diabetes: 5 year follow-up of an open-label, single-centre, randomised controlled trial. Lancet (2015) 386(9997):964–73. doi: 10.1016/S0140-6736(15)00075-6 26369473

[54] Sarma S Lipscombe LL. In obesity with T2DM, biliopancreatic diversion increased T2DM remission vs. medical and lifestyle therapy at 10 y. Ann Intern Med (2021) 174(6):JC70. doi: 10.7326/ACPJ202106150-070 34058110

[55] Michaud A Grenier-Larouche T Caron-Dorval D Marceau S Biertho L Simard S. Biliopancreatic diversion with duodenal switch leads to better postprandial glucose level and beta cell function than sleeve gastrectomy in individuals with type 2 diabetes very early after surgery. Metabolism (2017) 74:10–21. doi: 10.1016/j.metabol.2017.06.005 28764844

[56] McInnes N Hall S Lochnan HA Harris SB Punthakee Z Sigal RJ. REMIT-iGlarLixi Collaborative Group. Diabetes remission and relapse following an intensive metabolic intervention combining insulin glargine/lixisenatide, metformin and lifestyle approaches: Results of a randomised controlled trial. Diabetes Obes Metab (2023) 25(11):3347–55. doi: 10.1111/dom.15234 37580972

[57] Bohula EA Scirica BM Inzucchi SE McGuire DK Keech AC Smith SR. CAMELLIA-TIMI 61 Steering Committee Investigators. Effect of lorcaserin on prevention and remission of type 2 diabetes in overweight and obese patients (CAMELLIA-TIMI 61): a randomised, placebo-controlled trial. Lancet (2018) 392(10161):2269–79. doi: 10.1016/S0140-6736(18)32328-6 30293771

[58] Saiki A Yamaguchi T Sasaki A Naitoh T Matsubara H Yokote K. Background characteristics and diabetes remission after laparoscopic sleeve gastrectomy in Japanese patients with type 2 diabetes stratified by BMI: subgroup analysis of J-SMART. Diabetol Int (2021) 12(3):303–12. doi: 10.1007/s13340-020-00487-x PMC8172703 34150439

[59] Ohira M Watanabe Y Yamaguchi T Saiki A Nakamura S Tanaka S. Determinants of type 2 diabetes remission after bariatric surgery in obese Japanese patients: a retrospective cohort study. Diabetol Int (2021) 12(4):379–88. doi: 10.1007/s13340-021-00493-7 PMC8413414 34567920

[60] Mateo-Gavira I Sánchez-Toscano E Mayo-Ossorio MÁ Pacheco-García JM Prada-Oliveira JA Vílchez-López FJ. Evaluation of clinical factors predictive of diabetes remission following bariatric surgery. J Clin Med (2021) 10(9):1945. doi: 10.3390/jcm10091945 34062745 PMC8124312

[61] Gutierrez-Mariscal FM Alcalá-Diaz JF Quintana-Navarro GM de la Cruz-Ares S Torres-Peña JD Cardelo MP. Changes in quantity plant-based protein intake on type 2 diabetes remission in coronary heart disease patients: from the CORDIOPREV study. Eur J Nutr (2023) 62(4):1903–13. doi: 10.1007/s00394-022-03080-x PMC10195707 36869909

[62] Chandru S Pramodkumar TA Pradeepa R Muthukumar S Balasubramanyam M Bhuvaneshwari R. Outcomes of metabolic surgery in obese patients with type 2 diabetes with respect to impact on beta cell function, insulin sensitivity and diabetes remission - a study from south India. Diabetes Metab Syndr (2020) 14(6):1829–35. doi: 10.1016/j.dsx.2020.09.010 32961515

[63] Praveen Raj P Bhattacharya S Saravana Kumar S Sabnis SC Parthasarathi R Swamy PDK. Do bariatric surgery-related type 2 diabetes remission predictors add clinical value? A study on Asian Indian obese diabetics. Obes Surg (2017) 27(8):2113–9. doi: 10.1007/s11695-017-2615-8 28236254

[64] Madsen LR Espersen R Ornstrup MJ Jørgensen NR Langdahl BL Richelsen B. Bone health in patients with type 2 diabetes treated by Roux-en-Y gastric bypass and the role of diabetes remission. Obes Surg (2019) 29(6):1823–31. doi: 10.1007/s11695-019-03753-3 30719648

[65] Madsen LR Baggesen LM Richelsen B Thomsen RW. Effect of Roux-en-Y gastric bypass surgery on diabetes remission and complications in individuals with type 2 diabetes: a Danish population-based matched cohort study. Diabetologia (2019) 62(4):611–20. doi: 10.1007/s00125-019-4816-2 30734055

[66] Hao S Umpierrez GE Daley T Vellanki P. Intervention with therapeutic agents, understanding the path to remission in type 2 diabetes: part 1. Endocrinol Metab Clin North Am (2023) 52(1):27–38. doi: 10.1016/j.ecl.2022.07.003 36754495

[67] Dayan CM Korah M Tatovic D Bundy BN Herold KC. Changing the landscape for type 1 diabetes: the first step to prevention. Lancet (2019) 394(10205):1286–96. doi: 10.1016/S0140-6736(19)32127-0 31533907

[68] Gomez-Muñoz L Perna-Barrull D Caroz-Armayones JM Murillo M Rodriguez-Fernandez S Valls A. Candidate biomarkers for the prediction and monitoring of partial remission in pediatric type 1 diabetes. Front Immunol (2022) 13:825426. doi: 10.3389/fimmu.2022.825426 35280980 PMC8904370

[69] Wan XX Zhang DY Khan MA Zheng SY Hu XM Zhang Q. Stem cell transplantation in the treatment of type 1 diabetes mellitus: from insulin replacement to beta-cell replacement. Front Endocrinol (Lausanne) (2022) 13:859638. doi: 10.3389/fendo.2022.859638 35370989 PMC8972968

[70] Inaishi J Saisho Y. Beta-cell mass in obesity and type 2 diabetes, and its relation to pancreas fat: a mini-review. Nutrients (2020) 12(12):3846. doi: 10.3390/nu12123846 33339276 PMC7766247

[71] Lingvay I Sumithran P Cohen RV le Roux CW. Obesity management as a primary treatment goal for type 2 diabetes: time to reframe the conversation. Lancet (2022) 399(10322):394–405. doi: 10.1016/S0140-6736(21)01919-X 34600604

[72] Petrov MS Taylor R. Intra-pancreatic fat deposition: bringing hidden fat to the fore. Nat Rev Gastroenterol Hepatol (2022) 19(3):153–68. doi: 10.1038/s41575-021-00551-0 34880411

[73] Meerasa A Dash S. Weighing in on type 2 diabetes remission. Diabetes Care (2022) 45(1):28–30. doi: 10.2337/dci21-0041 34986262

[74] Barthold D Brouwer E Barton LJ Arterburn DE Basu A Courcoulas A. Minimum threshold of bariatric surgical weight loss for initial diabetes remission. Diabetes Care (2022) 45(1):92–9. doi: 10.2337/dc21-0714 PMC8753771 34518376

[75] Kelly WD Lillehei RC Merkel FK Idezuki Y Goetz FC. Allotransplantation of the pancreas and duodenum along with the kidney in diabetic nephropathy. Surgery (1967) 61(6):827–37. 5338113

[76] Ramzy A Belmonte PJ Braam MJS Ida S Wilts EM Levings MK. A century-long journey from the discovery of insulin to the Iimplantation of stem cell-derived islets. Endocr Rev (2023) 44(2):222–53. doi: 10.1210/endrev/bnac021 36111962

[77] McCarthy M. Canadian group reports best results yet with islet-cell transplants. Lancet (2000) 355(9221):2140. doi: 10.1016/s0140-6736(05)72777-x 10902636

[78] Marfil-Garza BA Imes S Verhoeff K Hefler J Lam A Dajani K. Pancreatic islet transplantation in type 1 diabetes: 20-year experience from a single-centre cohort in Canada. Lancet Diabetes Endocrinol (2022) 10(7):519–32. doi: 10.1016/S2213-8587(22)00114-0 35588757

[79] Marfil-Garza BA Hefler J Verhoeff K Lam A Dajani K Anderson B. Pancreas and islet transplantation: comparative outcome analysis of a single-centre cohort over 20-years. Ann Surg (2023) 277(4):672–80. doi: 10.1097/SLA.0000000000005783 36538619

[80] Shapiro AM Ricordi C Hering BJ Auchincloss H Lindblad R Robertson RP. International trial of the Edmonton protocol for islet transplantation. N Engl J Med (2006) 355(13):1318–30. doi: 10.1056/NEJMoa061267 17005949

[81] Ryan EA Lakey JR Rajotte RV Korbutt GS Kin T Imes S. Clinical outcomes and insulin secretion after islet transplantation with the Edmonton protocol. Diabetes (2001) 50(4):710–9. doi: 10.2337/diabetes.50.4.710 11289033

[82] Killestein J. Anti-CD3 monoclonal antibody in new-onset type 1 diabetes mellitus. N Engl J Med (2002) 347(14):1116–7. doi: 10.1056/NEJM200210033471416 12362018

[83] Ryu S Kodama S Ryu K Schoenfeld DA Faustman DL. Reversal of established autoimmune diabetes by restoration of endogenous beta cell function. J Clin Invest (2001) 108(1):63–72. doi: 10.1172/JCI12335 11435458 PMC209340

[84] Kojima H Fujimiya M Matsumura K Younan P Imaeda H Maeda M. NeuroD-betacellulin gene therapy induces islet neogenesis in the liver and reverses diabetes in mice. Nat Med (2003) 9(5):596–603. doi: 10.1038/nm867 12704384

[85] Kodama S Kühtreiber W Fujimura S Dale EA Faustman DL. Islet regeneration during the reversal of autoimmune diabetes in NOD mice. Science (2003) 302(5648):1223–7. doi: 10.1126/science.1088949 14615542

[86] Ryan EA Paty BW Senior PA Lakey JR Bigam D Shapiro AM. Beta-score: an assessment of beta-cell function after islet transplantation. Diabetes Care (200) 28(2):343–7. doi: 10.2337/diacare.28.2.343 15677790

[87] Atkinson MA. ADA outstanding scientific achievement lecture 2004. Thirty years of investigating the autoimmune basis for type 1 diabetes: why can't we prevent or reverse this disease? Diabetes (2005) 54(5):1253–63. doi: 10.2337/diabetes.54.5.1253 15855308

[88] D'Amour KA Bang AG Eliazer S Kelly OG Agulnick AD Smart NG. Production of pancreatic hormone-expressing endocrine cells from human embryonic stem cells. Nat Biotechnol (2006) 24(11):1392–401. doi: 10.1038/nbt1259 17053790

[89] Kramer CK Zinman B Retnakaran R. Short-term intensive insulin therapy in type 2 diabetes mellitus: a systematic review and meta-analysis. Lancet Diabetes Endocrinol (2013) 1(1):28–34. doi: 10.1016/S2213-8587(13)70006-8 24622264

[90] Kramer CK Zinman B Choi H Retnakaran R. Predictors of sustained drug-free diabetes remission over 48 weeks following short-term intensive insulin therapy in early type 2 diabetes. BMJ Open Diabetes Res Care (2016) 4(1):e000270. doi: 10.1136/bmjdrc-2016-000270 PMC4985916 27547422

[91] Rubino F Nathan DM Eckel RH Schauer PR Alberti KG Zimmet PZ. Delegates of the 2nd Diabetes Surgery Summit. Metabolic surgery in the treatment algorithm for type 2 diabetes: a joint statement by international diabetes organizations. Diabetes Care (2016) 39(6):861–77. doi: 10.2337/dc16-0236 27222544

[92] Latteri S Sofia M Puleo S Di Vincenzo A Cinti S Castorina S. Mechanisms linking bariatric surgery to adipose tissue, glucose metabolism, fatty liver disease and gut microbiota. Langenbecks Arch Surg (2023) 408(1):101. doi: 10.1007/s00423-023-02821-8 36826628 PMC9957865

[93] de Abreu Sesconetto L da Silva RBR Galletti RP Agareno GA Colonno BB de Sousa JHB. Scores for predicting diabetes remission in bariatric surgery: a systematic review and meta-analysis. Obes Surg (2023) 33(2):600–10. doi: 10.1007/s11695-022-06382-5 36456846

[94] Brown A McArdle P Taplin J Unwin D Unwin J Deakin T. Dietary strategies for remission of type 2 diabetes: A narrative review. J Hum Nutr Diet (2022) 35(1):165–78. doi: 10.1111/jhn.12938 34323335

[95] Kelly J Karlsen M Steinke G. Type 2 diabetes remission and lifestyle medicine: a position statement from the American College of Lifestyle Medicine. Am J Lifestyle Med (2020) 14(4):406–19. doi: 10.1177/1559827620930962 PMC7692017 33281521

[96] Rosenfeld RM Kelly JH Agarwal M Aspry K Barnett T Davis BC. Dietary interventions to treat type 2 diabetes in adults with a goal of remission: an expert consensus statement from the American College of Lifestyle Medicine. Am J Lifestyle Med (2022) 16(3):342–62. doi: 10.1177/15598276221087624 PMC9189586 35706589

[97] Ko JH Kim TN. Type 2 diabetes remission with significant weight loss: definition and evidence-based interventions. J Obes Metab Syndr (2022) 31:123–33. doi: 10.7570/jomes22001 PMC9284579 35618657

[98] Banerjee ES Schroeder R Harrison TD. Metabolic surgery for adult obesity: common questions and answers. Am Fam Phys (2022) 105:593–601. 35704821

[99] Li Y Xu W Liao Z Yao B Chen X Huang Z. Induction of long-term glycemic control in newly diagnosed type 2 diabetic patients is associated with improvement of beta-cell function. Diabetes Care (2004) 27:2597–602. doi: 10.2337/diacare.27.11.2597 15504992

[100] Chen HS Wu TE Kuo CS. Long-term glycemic control after 6 months of basal insulin therapy. Am J Manag Care (2014) 20(9):e369–79. 25364873

[101] Nanna MG Kolkailah AA Page C Peterson ED Navar AM. Use of sodium-glucose cotransporter 2 inhibitors and glucagonlike peptide-1 receptor agonists in patients with diabetes and cardiovascular disease in community practice. JAMA Cardiol (2023) 8(1):89–95. doi: 10.1001/jamacardio.2022.3839 36322056 PMC9631221

[102] Kotit S. EMMY: The continued expansion of clinical applications of SGLT2 inhibitors. Glob Cardiol Sci Pract (2023) 2023(1):e202305. doi: 10.21542/gcsp.2023.5 36890845 PMC9988294

[103] Ma H Lin YH Dai LZ Lin CS Huang Y Liu SY. Efficacy and safety of GLP-1 receptor agonists versus SGLT-2 inhibitors in overweight/obese patients with or without diabetes mellitus: a systematic review and network meta-analysis. BMJ Open (2023) 13(3):e061807. doi: 10.1136/bmjopen-2022-061807 PMC10008474 36882248

[104] Aldawsari M Almadani FA Almuhammadi N Algabsani S Alamro Y Aldhwayan M. The efficacy of GLP-1 analogues on appetite parameters, gastric emptying, food preference and taste among adults with obesity: systematic review of randomized controlled trials. Diabetes Metab Syndr Obes (2023) 16:575–95. doi: 10.2147/DMSO.S387116 PMC9987242 36890965

[105] Naseralallah L Aboujabal B. Profile of tirzepatide in the management of type 2 diabetes mellitus: design, development, and place in therapy. Expert Opin Pharmacother (2023) 24(4):407–18. doi: 10.1080/14656566.2023.2181074 36820516

[106] Jiang L Zhang Y Zhang H Chen Y Huang W Xiao Y. Comparative efficacy of 6 traditional Chinese patent medicines combined with lifestyle modification in patients with prediabetes: A network meta-analysis. Diabetes Res Clin Pract (2022) 188:109878. doi: 10.1016/j.diabres.2022.109878 35483544

